# Supplementary material for: The rhythms of the night: increase in online night activity and emotional resilience during the spring 2020 Covid-19 lockdown
Source: EPJ Data Sci. 2021 Feb 1;10(1):7. doi: 10.1140/epjds/s13688-021-00262-1 (PMC7848867; doi:10.1140/epjds/s13688-021-00262-1)
Supplement: Supplementary file 1 — Supplementary information (PDF 1.5 MB) [file 13688_2021_262_MOESM1_ESM.pdf]

Supplementary Material:
list of the analyzed YouTube channels

channel	chan_title	url
**-2EkisRV8h9KsHpslQ1gXA**	Et tout le monde s'en fout	https://www.youtube.com/channel/-2EkisRV8h9KsHpslQ1gXA
**-4WUubuVGowG_R7gdgesPA**	Max Bird	https://www.youtube.com/channel/-4WUubuVGowG_R7gdgesPA
**-5QzbwkKnohEZPcrc4NjJQ**	BioticTV	https://www.youtube.com/channel/-5QzbwkKnohEZPcrc4NjJQ
**-B-ngGIHiiAHNDeqeJgXFA**	ERTV Rhône-Alpes	https://www.youtube.com/channel/-B-ngGIHiiAHNDeqeJgXFA
**-KZ5gLMqOAtFMxoWz03kPA**	Corinne Vignon	https://www.youtube.com/channel/-KZ5gLMqOAtFMxoWz03kPA
**-OKvAo7bzZTAkqbPS9eLiQ**	LIB TROPIQUES	https://www.youtube.com/channel/-OKvAo7bzZTAkqbPS9eLiQ
**-P--TViDm4ZdCPIxFw0mzg**	Natixisvideos	https://www.youtube.com/channel/-P--TViDm4ZdCPIxFw0mzg
**-UfDZOxYSEH705uHiTQLkQ**	MrMumudu56	https://www.youtube.com/channel/-UfDZOxYSEH705uHiTQLkQ
**-V7Kcar0M76Xc1iHqPuUSw**	CDI Médias	https://www.youtube.com/channel/-V7Kcar0M76Xc1iHqPuUSw
**-X7w2qqOylWyJ0Q471_Jpg**	Députée Géraldine Bannier	https://www.youtube.com/channel/-X7w2qqOylWyJ0Q471_Jpg
**-diLiHNfmmninG_OKkql3w**	Xavier Breton	https://www.youtube.com/channel/-diLiHNfmmninG_OKkql3w
**-e0hilusTvqgqfCgLyGBnQ**	Leïla Chaibi	https://www.youtube.com/channel/-e0hilusTvqgqfCgLyGBnQ
**-el1KT7oSSqHICexbdpfpg**	Fan D'humour	https://www.youtube.com/channel/-el1KT7oSSqHICexbdpfpg
**-g3axVNt-E92HJdX2Bd9gA**	AMAL AMELIA LAKRAFI - FDE CIRCO 10	https://www.youtube.com/channel/-g3axVNt-E92HJdX2Bd9gA
**-hrZHSpKnCwH7tSRRJ2pVA**	Grand Lyon TV	https://www.youtube.com/channel/-hrZHSpKnCwH7tSRRJ2pVA
**-zV7P-dlOAZefo4_HKAX7g**	street 45	https://www.youtube.com/channel/-zV7P-dlOAZefo4_HKAX7g
**0--537XHg1DCMgU9a1sHxQ**	agenceinfolibre	https://www.youtube.com/channel/0--537XHg1DCMgU9a1sHxQ
**066ZLZxX7S0UyKJB5nDR_Q**	Clicanoo	https://www.youtube.com/channel/066ZLZxX7S0UyKJB5nDR_Q
**0HxyEc_ojRJ1oJXS5K6oaA**	Politikon	https://www.youtube.com/channel/0HxyEc_ojRJ1oJXS5K6oaA
**0Uge1s4twAaYf_nb9WPZkQ**	ATSADA Arezki	https://www.youtube.com/channel/0Uge1s4twAaYf_nb9WPZkQ
**0VWNduqFrhajYPI7Z6aMgw**	Pleinchamp	https://www.youtube.com/channel/0VWNduqFrhajYPI7Z6aMgw
**0Y9vEv67BYRhgbRwJGj35w**	Ministère des Armées	https://www.youtube.com/channel/0Y9vEv67BYRhgbRwJGj35w
**0cT8BZdFUmC0vgr1XS7aPA**	Tatiana Ventôse	https://www.youtube.com/channel/0cT8BZdFUmC0vgr1XS7aPA
**0gI048KH8MB8PxrMMVPzjg**	Panamza	https://www.youtube.com/channel/0gI048KH8MB8PxrMMVPzjg
**0i7t1CC7T0xheeBahaWZYQ**	Clément MONTFORT - Web-Séries Documentaires	https://www.youtube.com/channel/0i7t1CC7T0xheeBahaWZYQ
**0qfZ-P0THJtc2TUPzUGiVQ**	Alexandre Holroyd	https://www.youtube.com/channel/0qfZ-P0THJtc2TUPzUGiVQ
**0suOBgRAZPCy5kD6IS23zw**	Pierre Chevelle - Changer le monde en 2 heures	https://www.youtube.com/channel/0suOBgRAZPCy5kD6IS23zw
**0t5smuKObeRPD7lW2cZp-g**	HORS-ZONE Press	https://www.youtube.com/channel/0t5smuKObeRPD7lW2cZp-g
**0yPCUmdMZIGtnxSnx5_ifA**	Tzitzimitl - Esprit Critique	https://www.youtube.com/channel/0yPCUmdMZIGtnxSnx5_ifA
**1-tuwsZmt24TVjqb2NUD3w**	TV JE SUIS BLOCUS	https://www.youtube.com/channel/1-tuwsZmt24TVjqb2NUD3w
**11tPrPcudLv8J2ladG2DsA**	Hors-Série	https://www.youtube.com/channel/11tPrPcudLv8J2ladG2DsA
**12YKQ5OG-vsWIiIxjbDYVA**	Bourquin Jean-Charles	https://www.youtube.com/channel/12YKQ5OG-vsWIiIxjbDYVA
**191b1eOnxLhFb2cNLCpH7Q**	Rav DynoviszTV	https://www.youtube.com/channel/191b1eOnxLhFb2cNLCpH7Q
**1EacOJoqsKaYxaDomTCTEQ**	Le Réveilleur	https://www.youtube.com/channel/1EacOJoqsKaYxaDomTCTEQ
**1Gn800It-G7OIC4TOr0cIg**	PaulSereOfficiel	https://www.youtube.com/channel/1Gn800It-G7OIC4TOr0cIg
**1JEQiM754n4EYg2b_63zpw**	keopi cat	https://www.youtube.com/channel/1JEQiM754n4EYg2b_63zpw
**1K5y0hePYbnRDmKOMPo2JA**	melty	https://www.youtube.com/channel/1K5y0hePYbnRDmKOMPo2JA
**1ObaaFz4XHVPN2T5IFsU4w**	L'Obs	https://www.youtube.com/channel/1ObaaFz4XHVPN2T5IFsU4w
**1ZSjEKLP0PiB_BP2U8MhGw**	Sortez de la Matrice, la chaîne de Vahine	https://www.youtube.com/channel/1ZSjEKLP0PiB_BP2U8MhGw
**1h7WA1EXc8VVr-DGU0eqKA**	Valéry Coquant	https://www.youtube.com/channel/1h7WA1EXc8VVr-DGU0eqKA
**1isEuz-3EJ_U8soaUi00HA**	Danielle Simonnet	https://www.youtube.com/channel/1isEuz-3EJ_U8soaUi00HA
**1mB5ZW11oqDD1hO4uTH2lw**	bjp abadie	https://www.youtube.com/channel/1mB5ZW11oqDD1hO4uTH2lw
**1tFkn2ifypmHfGDLsB7Vpw**	Frédérick Moulin	https://www.youtube.com/channel/1tFkn2ifypmHfGDLsB7Vpw
**1yLBhh8eDSPm-_Hgz8AvNA**	Slate.fr	https://www.youtube.com/channel/1yLBhh8eDSPm-_Hgz8AvNA
**20dgIUTkVqzY9ipx_iOnng**	France 3 Occitanie	https://www.youtube.com/channel/20dgIUTkVqzY9ipx_iOnng
**287q-rLYtEuL2VZF5ag_ug**	actuman	https://www.youtube.com/channel/287q-rLYtEuL2VZF5ag_ug
**2CiAQw-MS0pZcept0Q152g**	WhataBeautifulWorld Youtube Channel	https://www.youtube.com/channel/2CiAQw-MS0pZcept0Q152g
**2EKIGQj0yijoc0H8D0DRcQ**	Les infos d'Alex	https://www.youtube.com/channel/2EKIGQj0yijoc0H8D0DRcQ
**2PAw2DM5hDTO-6CS7JjZMQ**	Line Press	https://www.youtube.com/channel/2PAw2DM5hDTO-6CS7JjZMQ
**2Q5phDGoRgl3WIR24PxmCw**	Serviteur de paix	https://www.youtube.com/channel/2Q5phDGoRgl3WIR24PxmCw
**2avy3Pwc3yVtt-tPKJn33A**	ma ferme autonome	https://www.youtube.com/channel/2avy3Pwc3yVtt-tPKJn33A

**2ezfc64OTaeobrKC0ZT-2A**	Objectif Gard	https://www.youtube.com/channel/2ezfc64OTaeobrKC0ZT-2A
**2g8dbjgwzOoBStMbar6wCQ**	EuropeIsrael	https://www.youtube.com/channel/2g8dbjgwzOoBStMbar6wCQ
**2i-BljAg1w_MMMA1JuFm7w**	La Provence	https://www.youtube.com/channel/2i-BljAg1w_MMMA1JuFm7w
**2j8KuuHh6EgUr4o9qgKisw**	Media Investigation	https://www.youtube.com/channel/2j8KuuHh6EgUr4o9qgKisw
**2kC77turr-0q-idWYrl1YA**	INSOLENTIAE TV	https://www.youtube.com/channel/2kC77turr-0q-idWYrl1YA
**2kIberIFY054MWlDWNebxA**	Mouton Lucide	https://www.youtube.com/channel/2kIberIFY054MWlDWNebxA
**2uPHgyyLv2rnkm4CfIgWcw**	MisterJDay	https://www.youtube.com/channel/2uPHgyyLv2rnkm4CfIgWcw
**2vsKHUbDUOg02gLxLoxplA**	Paula Forteza	https://www.youtube.com/channel/2vsKHUbDUOg02gLxLoxplA
**2wrXSZMid7OiCVwCfVpHiA**	Lundi Matin	https://www.youtube.com/channel/2wrXSZMid7OiCVwCfVpHiA
**38_Fg9HQ5HsPHX6U5y239g**	lesurvivaliste	https://www.youtube.com/channel/38_Fg9HQ5HsPHX6U5y239g
**38xK7hksnyzdWPdf0XC9kQ**	Artémisia Collège	https://www.youtube.com/channel/38xK7hksnyzdWPdf0XC9kQ
**3BW_IlgG6orWiej8jYpEow**	Académie des sciences	https://www.youtube.com/channel/3BW_IlgG6orWiej8jYpEow
**3E2DhYIqnoc6H3WXwTVnlA**	Le blob, l'extra-média	https://www.youtube.com/channel/3E2DhYIqnoc6H3WXwTVnlA
**3FCKjZ4X77uJB7WSQfqk_g**	Damoclès France	https://www.youtube.com/channel/3FCKjZ4X77uJB7WSQfqk_g
**3FUHIUhkXVRS8PQgheGz4A**	BTLV Le média complémentaire	https://www.youtube.com/channel/3FUHIUhkXVRS8PQgheGz4A
**3HIHM5TmtTzVZPNjWP22sA**	SYMPA TIK	https://www.youtube.com/channel/3HIHM5TmtTzVZPNjWP22sA
**3Ma4tRFxx85oZI_XKVTPwg**	les Républicains	https://www.youtube.com/channel/3Ma4tRFxx85oZI_XKVTPwg
**3Mj5hKC0Nbqv3YXHLKxdyw**	Studio Crapulax	https://www.youtube.com/channel/3Mj5hKC0Nbqv3YXHLKxdyw
**3QEfPR2uqpn4uZmaUMyqPQ**	Genco	https://www.youtube.com/channel/3QEfPR2uqpn4uZmaUMyqPQ
**3RfG8bCbXR-Ae9iVHqXtTQ**	Marie S'Infiltre	https://www.youtube.com/channel/3RfG8bCbXR-Ae9iVHqXtTQ
**3RlAzNzgy_7QiVPukFiDZQ**	chris la parole	https://www.youtube.com/channel/3RlAzNzgy_7QiVPukFiDZQ
**3a0sxZ00y193_aKpbfWYpA**	Nico & Mariana	https://www.youtube.com/channel/3a0sxZ00y193_aKpbfWYpA
**3fXXuC9sR7jNynrPUFFnAA**	Demo Sophie	https://www.youtube.com/channel/3fXXuC9sR7jNynrPUFFnAA
**3q3FLPQtuWWv1uTkTfpEtw**	LE BON SENS	https://www.youtube.com/channel/3q3FLPQtuWWv1uTkTfpEtw
**3xsncRKW4hNVgx-E5Y3qvw**	Factuelle 66	https://www.youtube.com/channel/3xsncRKW4hNVgx-E5Y3qvw
**45NsEyqTGMtOeEM-UvlTuQ**	Regards	https://www.youtube.com/channel/45NsEyqTGMtOeEM-UvlTuQ
**48e1bQSz_FYKSqIPjZW_BA**	Permanence de Didier Le Gac	https://www.youtube.com/channel/48e1bQSz_FYKSqIPjZW_BA
**4A05JQhUDYjFJA8VtVXJbQ**	DE QUOI ON CAUSE ?	https://www.youtube.com/channel/4A05JQhUDYjFJA8VtVXJbQ
**4A3K4CT4swDAW57fzXIMVA**	Eric Coquerel	https://www.youtube.com/channel/4A3K4CT4swDAW57fzXIMVA
**4BQzxhnIvEvOr-385pwK_Q**	C8	https://www.youtube.com/channel/4BQzxhnIvEvOr-385pwK_Q
**4Bw8KLhbLPrcZbOAJa4p0w**	Ina Actu	https://www.youtube.com/channel/4Bw8KLhbLPrcZbOAJa4p0w
**4CsR6zpmNlId_zz61CKMVg**	CHL.TV	https://www.youtube.com/channel/4CsR6zpmNlId_zz61CKMVg
**4Ds3UCi1pAUFEPss9NH28Q**	Sylvain Maillard	https://www.youtube.com/channel/4Ds3UCi1pAUFEPss9NH28Q
**4EgWvItklyJ55cj-9dIsKQ**	Miny Nas	https://www.youtube.com/channel/4EgWvItklyJ55cj-9dIsKQ
**4Euikzr0bLKsnNuIKW6Sfg**	On n'est pas couché	https://www.youtube.com/channel/4Euikzr0bLKsnNuIKW6Sfg
**4FlaaYbbdizLZUo_h_UzGw**	Dominique Potier	https://www.youtube.com/channel/4FlaaYbbdizLZUo_h_UzGw
**4IoY08ud4Mi_tP9abGBk2A**	L'Effet Chimpanzé	https://www.youtube.com/channel/4IoY08ud4Mi_tP9abGBk2A
**4Ku_yoH9xNxU5Nn5ly7XAQ**	Vox Pop - ARTE	https://www.youtube.com/channel/4Ku_yoH9xNxU5Nn5ly7XAQ
**4LADtN8xwppFydHUB1s5uA**	Cosmopolitan	https://www.youtube.com/channel/4LADtN8xwppFydHUB1s5uA
**4OHk_5MYu_QZ5HQmUUzUYQ**	jayslem jayslem	https://www.youtube.com/channel/4OHk_5MYu_QZ5HQmUUzUYQ
**4Q2EebrNVAbB6w4PnFGmyw**	Vik Gadsden	https://www.youtube.com/channel/4Q2EebrNVAbB6w4PnFGmyw
**4V128uqm6Ui9kBQcQisi-w**	Union des Démocrates Musulmans Français	https://www.youtube.com/channel/4V128uqm6Ui9kBQcQisi-w
**4e6cfcOXGycjRLZbbSwwoA**	Ville de Paris	https://www.youtube.com/channel/4e6cfcOXGycjRLZbbSwwoA
**4ii4_aeS8iOFzsHuhJTq2w**	Poisson Fécond	https://www.youtube.com/channel/4ii4_aeS8iOFzsHuhJTq2w
**4juGR_3VodUrCOn7hCVsTQ**	Eric Plisson	https://www.youtube.com/channel/4juGR_3VodUrCOn7hCVsTQ
**4kluHyo3ROaIKuXmQ1YupQ**	Armée française - Opérations militaires	https://www.youtube.com/channel/4kluHyo3ROaIKuXmQ1YupQ
**4pqBlgpmRCMtjAxiKq4OiA**	logan moreno	https://www.youtube.com/channel/4pqBlgpmRCMtjAxiKq4OiA
**4rdaBAEFlK7NXhSkb3UhpQ**	petiplus toulousain	https://www.youtube.com/channel/4rdaBAEFlK7NXhSkb3UhpQ
**50uCdUubc04xXZz7bqv5ng**	Investigations et Enquêtes	https://www.youtube.com/channel/50uCdUubc04xXZz7bqv5ng
**52yOGvUSATyZdjL_2-DehA**	lejournaldepersonne	https://www.youtube.com/channel/52yOGvUSATyZdjL_2-DehA
**53gfTiWvslLPNuoDcoxmVg**	Franjo	https://www.youtube.com/channel/53gfTiWvslLPNuoDcoxmVg
**56fVrfUL_1yF2EXMU9qCoA**	France Bleu Paris et sa région	https://www.youtube.com/channel/56fVrfUL_1yF2EXMU9qCoA
**57sZH8K-B0yHjnNsfJHvMA**	Pompiers de Paris	https://www.youtube.com/channel/57sZH8K-B0yHjnNsfJHvMA
**585x08xU5MlwnwgowaPLCQ**	J'ai Un Doute	https://www.youtube.com/channel/585x08xU5MlwnwgowaPLCQ

**59NZI9h9pWQON3WlANJaLw**	Corse Net Infos	https://www.youtube.com/channel/59NZI9h9pWQON3WlANJaLw
**5COTnDgJep08Blz-IJqk_Q**	Martial Ier Gueux.0	https://www.youtube.com/channel/5COTnDgJep08Blz-IJqk_Q
**5HYOg4Qybf-T_NFa4FP3CQ**	Actu Médias	https://www.youtube.com/channel/5HYOg4Qybf-T_NFa4FP3CQ
**5RNdOTEAv8YaRQESIDS08w**	JE M'INFORME TV	https://www.youtube.com/channel/5RNdOTEAv8YaRQESIDS08w
**5WjQI_NY-PODOQ3kTewebA**	France Bleu	https://www.youtube.com/channel/5WjQI_NY-PODOQ3kTewebA
**5X4e8ScZI2AFd_vkjSoyoQ**	AstronoGeek	https://www.youtube.com/channel/5X4e8ScZI2AFd_vkjSoyoQ
**5a1vcRZdol1qIRRdQoEX6g**	Planetes360	https://www.youtube.com/channel/5a1vcRZdol1qIRRdQoEX6g
**5dJGghNJhZmVrdZ3cCWMgw**	Maison départementale de l'environnement du Mais	https://www.youtube.com/channel/5dJGghNJhZmVrdZ3cCWMgw
**5eOLQO5VUEFJukNg9cl5jg**	Histoire Brève	https://www.youtube.com/channel/5eOLQO5VUEFJukNg9cl5jg
**5g0EgB5QTjp8NQPl61rSAg**	Jacques Marilossian	https://www.youtube.com/channel/5g0EgB5QTjp8NQPl61rSAg
**5hVF83yZck7wWp78ffFYPQ**	PhilippeBuffon	https://www.youtube.com/channel/5hVF83yZck7wWp78ffFYPQ
**5kLs1gGQ6NRHzM5CNourdg**	Zinfos974	https://www.youtube.com/channel/5kLs1gGQ6NRHzM5CNourdg
**5pwpz5QtTlW4uIFyDnugqA**	Paul Jorion	https://www.youtube.com/channel/5pwpz5QtTlW4uIFyDnugqA
**5s7HmoOXXIc1Z-Rlz4FAcA**	NOUS VOULONS VIVRE !	https://www.youtube.com/channel/5s7HmoOXXIc1Z-Rlz4FAcA
**5yfUEvxrnrj4Gyr0evSV-w**	UFC-Que Choisir	https://www.youtube.com/channel/5yfUEvxrnrj4Gyr0evSV-w
**5z-dw1g1G2_twr2-RAtdHQ**	Entracte Science	https://www.youtube.com/channel/5z-dw1g1G2_twr2-RAtdHQ
**6-BWVphnrCj5xNL73qOd0w**	Cemil Choses A Te Dire	https://www.youtube.com/channel/6-BWVphnrCj5xNL73qOd0w
**62CU0yy2M0hSddi9asTikg**	Pau Béarn Pyrénées	https://www.youtube.com/channel/62CU0yy2M0hSddi9asTikg
**69rAVbyGfQMa2EALOfho_w**	Treize T.D.L. territoire des loups	https://www.youtube.com/channel/69rAVbyGfQMa2EALOfho_w
**6AiVZlJJMXo7aAtpWdhapA**	patrick demoulin	https://www.youtube.com/channel/6AiVZlJJMXo7aAtpWdhapA
**6BAWsOh62mX32azpynAgOA**	Easy Predictions	https://www.youtube.com/channel/6BAWsOh62mX32azpynAgOA
**6Ba7StC1ervhrpH_ozhR3A**	InfoCritiqueWeb	https://www.youtube.com/channel/6Ba7StC1ervhrpH_ozhR3A
**6CeKvArSXPA3VJ2VU5vO6A**	Yves Barraud	https://www.youtube.com/channel/6CeKvArSXPA3VJ2VU5vO6A
**6QD7eTqSxDy58Dxi4CaU5Q**	La Chronique Politique	https://www.youtube.com/channel/6QD7eTqSxDy58Dxi4CaU5Q
**6bnDZPS-B-e0mDz7a9g9UA**	Paul disciple du Christ	https://www.youtube.com/channel/6bnDZPS-B-e0mDz7a9g9UA
**6bn_ABjeTwbJFGFMQbZFlw**	Loopsider	https://www.youtube.com/channel/6bn_ABjeTwbJFGFMQbZFlw
**6c9cFLIKc7Kakv0dSzleVA**	Groupe Revue Fiduciaire	https://www.youtube.com/channel/6c9cFLIKc7Kakv0dSzleVA
**6dMVNhZmJYFf7SPv1WLMuQ**	Nat Geo France	https://www.youtube.com/channel/6dMVNhZmJYFf7SPv1WLMuQ
**6evEAa--4i0Mwf2QVA68JA**	Manifestons!	https://www.youtube.com/channel/6evEAa--4i0Mwf2QVA68JA
**6fppuwTtpcTERAsbCXcnJw**	Deborah Donnier	https://www.youtube.com/channel/6fppuwTtpcTERAsbCXcnJw
**6hR6KtxdA_6H_cE1HQIlbg**	Stéphane Edouard	https://www.youtube.com/channel/6hR6KtxdA_6H_cE1HQIlbg
**6i6RCczLaW9VVito-689IA**	Les Pensées de Riles	https://www.youtube.com/channel/6i6RCczLaW9VVito-689IA
**6joDErOGcFJC1vHNSfsYQg**	01net	https://www.youtube.com/channel/6joDErOGcFJC1vHNSfsYQg
**6kvMQP9gzv6qwwzMLuwZTg**	France 3 Grand Est	https://www.youtube.com/channel/6kvMQP9gzv6qwwzMLuwZTg
**6p_xVUVNX1HTMqGQgQvphQ**	j21unis	https://www.youtube.com/channel/6p_xVUVNX1HTMqGQgQvphQ
**6qRHsISN_A-hH0XNB2XOaQ**	Islamotion	https://www.youtube.com/channel/6qRHsISN_A-hH0XNB2XOaQ
**6thwlu5kQxznuGhurKshyQ**	Fabien GOUTTEFARDE	https://www.youtube.com/channel/6thwlu5kQxznuGhurKshyQ
**6tq7kO39mt9OY2BDS_oj0A**	NIAVLYS	https://www.youtube.com/channel/6tq7kO39mt9OY2BDS_oj0A
**6uo3uVzDvJVzrJ2DdmZT5Q**	Jean-Yves Le Gallou	https://www.youtube.com/channel/6uo3uVzDvJVzrJ2DdmZT5Q
**70XyVJcuRZlEMr7aQt_uKg**	ActuTV Actualités et Vidéos	https://www.youtube.com/channel/70XyVJcuRZlEMr7aQt_uKg
**71QbhNwsB7H5TASQSwCEiA**	maCommune.info	https://www.youtube.com/channel/71QbhNwsB7H5TASQSwCEiA
**72LKfVFMw_nXFzWhSQANlw**	zouloula100	https://www.youtube.com/channel/72LKfVFMw_nXFzWhSQANlw
**76K9ms1LM5uOQzOd424Oxw**	En marche avec Alain PEREA	https://www.youtube.com/channel/76K9ms1LM5uOQzOd424Oxw
**76pM1Auet3YolJ5vYLeINQ**	disclose ngo	https://www.youtube.com/channel/76pM1Auet3YolJ5vYLeINQ
**7FCUF2yedOT-Ot55kKZ1iw**	Veolia Group	https://www.youtube.com/channel/7FCUF2yedOT-Ot55kKZ1iw
**7G6uMPiyC8lV1IdUrMBB1w**	Département des Bouches-du-Rhône	https://www.youtube.com/channel/7G6uMPiyC8lV1IdUrMBB1w
**7HiAlb8wakDSKww5piOmkA**	Infos Toulouse	https://www.youtube.com/channel/7HiAlb8wakDSKww5piOmkA
**7OMeUFuZMacpPk2fc9UlOA**	Union Populaire Républicaine	https://www.youtube.com/channel/7OMeUFuZMacpPk2fc9UlOA
**7PX-myPpcT4YfZEWqZxGGg**	Sud Ouest	https://www.youtube.com/channel/7PX-myPpcT4YfZEWqZxGGg
**7Sc9GSh7ENQSszDybWPlWw**	Clarisse Delorient 2	https://www.youtube.com/channel/7Sc9GSh7ENQSszDybWPlWw
**7bu2tFVMExaDfZHNgmSOOA**	Ville de Nantes	https://www.youtube.com/channel/7bu2tFVMExaDfZHNgmSOOA
**7daY1GuhoUxgRhOcxP4SRA**	La vie est belle	https://www.youtube.com/channel/7daY1GuhoUxgRhOcxP4SRA
**7kNBYT4tg0OtciiZjQrV0g**	Institut de l'Economie Positive	https://www.youtube.com/channel/7kNBYT4tg0OtciiZjQrV0g
**7sFrgyGelEr6eny6_PsGXQ**	Tom's Guide France	https://www.youtube.com/channel/7sFrgyGelEr6eny6_PsGXQ

**7sXGI8p8PvKosLWagkK9wQ**	Heu?reka	https://www.youtube.com/channel/7sXGI8p8PvKosLWagkK9wQ
**7tPStufwtQk9-JHoo0TDig**	Journal Lamarseillaise	https://www.youtube.com/channel/7tPStufwtQk9-JHoo0TDig
**7vmFbbb2QxPIUhNQrQ3oEA**	Tv Languedoc	https://www.youtube.com/channel/7vmFbbb2QxPIUhNQrQ3oEA
**7xph2qAEotmyUPJbFVI95w**	Courrier international	https://www.youtube.com/channel/7xph2qAEotmyUPJbFVI95w
**83CfjOfvnJ8yuu9d_Ab5bw**	Philippe Michel-Kleisbauer	https://www.youtube.com/channel/83CfjOfvnJ8yuu9d_Ab5bw
**89C9Pjx5pAZgW6pBawPp9A**	EDF	https://www.youtube.com/channel/89C9Pjx5pAZgW6pBawPp9A
**8C4k0VR5msWubrhQMMy4xA**	Citoyen, citoyenne !	https://www.youtube.com/channel/8C4k0VR5msWubrhQMMy4xA
**8EzKGkEiusTm7g0mTfkWkg**	28 minutes - ARTE	https://www.youtube.com/channel/8EzKGkEiusTm7g0mTfkWkg
**8FnVm5FLHzs1uuhvIMgE_g**	Ville de Nice - Nice.fr tv	https://www.youtube.com/channel/8FnVm5FLHzs1uuhvIMgE_g
**8G4AdiEquZFrd0P_ZiW_rw**	Université d'Angers	https://www.youtube.com/channel/8G4AdiEquZFrd0P_ZiW_rw
**8Ii_Xrs5ETf8djMz9fCx2w**	Ramous	https://www.youtube.com/channel/8Ii_Xrs5ETf8djMz9fCx2w
**8KV2v9K8X6bMq1RfggUqzg**	dgfipmedia	https://www.youtube.com/channel/8KV2v9K8X6bMq1RfggUqzg
**8PLJgvC1VXzs5TpsTYc3Jg**	Liberté Egalité Fraternité Sinon C'est Dehors	https://www.youtube.com/channel/8PLJgvC1VXzs5TpsTYc3Jg
**8RvCAE_ub3uWq2leis4RLA**	frenchpatriot1006	https://www.youtube.com/channel/8RvCAE_ub3uWq2leis4RLA
**8T_vTz76WUsudvxPk6SLEw**	Un Monde Riant	https://www.youtube.com/channel/8T_vTz76WUsudvxPk6SLEw
**8UhS6US74TjA5ZyYxARP6w**	Fondation Brigitte Bardot	https://www.youtube.com/channel/8UhS6US74TjA5ZyYxARP6w
**8Ux-LOyEXeioYQ4LFzpBXw**	Aude WTFake	https://www.youtube.com/channel/8Ux-LOyEXeioYQ4LFzpBXw
**8Whw7rLqXvCLUhpjtGWbZw**	RING	https://www.youtube.com/channel/8Whw7rLqXvCLUhpjtGWbZw
**8YYzl8TwNkYMgb7WX0JDBA**	Toute la vérité	https://www.youtube.com/channel/8YYzl8TwNkYMgb7WX0JDBA
**8i5fABd505LQLaZtwyXIPg**	Video investigation	https://www.youtube.com/channel/8i5fABd505LQLaZtwyXIPg
**8jSdiTfai1PFwjQ9MrkkBg**	Géopolitis	https://www.youtube.com/channel/8jSdiTfai1PFwjQ9MrkkBg
**8o81D0CgSe-L6qyQ5DVx8A**	ODH Tv 2	https://www.youtube.com/channel/8o81D0CgSe-L6qyQ5DVx8A
**8oP9cR4TeUqDzrs1ry9BcQ**	Morgan Priest	https://www.youtube.com/channel/8oP9cR4TeUqDzrs1ry9BcQ
**8p3TbbKSWUUo1BYzhnAeGg**	Liberté Politique	https://www.youtube.com/channel/8p3TbbKSWUUo1BYzhnAeGg
**8xG8FJ6wYOmYczDF6ZZjLQ**	Futura	https://www.youtube.com/channel/8xG8FJ6wYOmYczDF6ZZjLQ
**90mP_TeDLziENuYCMxlfpw**	Armire	https://www.youtube.com/channel/90mP_TeDLziENuYCMxlfpw
**9BiNtgR44q9rmE1ybLXkiA**	Free Dom	https://www.youtube.com/channel/9BiNtgR44q9rmE1ybLXkiA
**9CNQgPaUY1GlnX_nLPtM-g**	Génération·s	https://www.youtube.com/channel/9CNQgPaUY1GlnX_nLPtM-g
**9ELDqvA7YZiVQ9tA0mDDqA**	jayslem officiel	https://www.youtube.com/channel/9ELDqvA7YZiVQ9tA0mDDqA
**9GGzAhhvhJO1hL10-BcgNA**	LeHuffPost	https://www.youtube.com/channel/9GGzAhhvhJO1hL10-BcgNA
**9JKcC-s_TX4jaOVitjECvg**	Brut.France	https://www.youtube.com/channel/9JKcC-s_TX4jaOVitjECvg
**9NB2nXjNtRabu3YLPB16Hg**	J'suis pas content TV	https://www.youtube.com/channel/9NB2nXjNtRabu3YLPB16Hg
**9U_1ONuDEj0ZfLXYFaTpVA**	Le Parti des Pas de Parti - Officiel	https://www.youtube.com/channel/9U_1ONuDEj0ZfLXYFaTpVA
**9faHFOrNIFxMXrpykPHOnA**	FRED	https://www.youtube.com/channel/9faHFOrNIFxMXrpykPHOnA
**9hHeywcPBnLglqnQRaNShQ**	Le Fil d'Actu - Officiel	https://www.youtube.com/channel/9hHeywcPBnLglqnQRaNShQ
**9n4DH9d58lgIUp5uDkoBww**	tvcarcassonne	https://www.youtube.com/channel/9n4DH9d58lgIUp5uDkoBww
**9sShhWss17hHmCdYTCh7Uw**	AUTOhebdo	https://www.youtube.com/channel/9sShhWss17hHmCdYTCh7Uw
**9tdrNLs9QpsFZI050UARkQ**	Armée de l'air	https://www.youtube.com/channel/9tdrNLs9QpsFZI050UARkQ
**9umvvn3t3pbwu6wCdSQHtw**	Gilets Jaunes Réveillés	https://www.youtube.com/channel/9umvvn3t3pbwu6wCdSQHtw
**A3dZqGawAvJO6Lexbj8EGg**	Le Monde Moderne	https://www.youtube.com/channel/A3dZqGawAvJO6Lexbj8EGg
**ABK_uTtowoJodTQVTN09uA**	Onze Mondial	https://www.youtube.com/channel/ABK_uTtowoJodTQVTN09uA
**ABqe4i9tBXzCgMpftEgaZw**	HENRY DE LESQUEN	https://www.youtube.com/channel/ABqe4i9tBXzCgMpftEgaZw
**AKbi-v4SkikcfjjQsGw-rA**	DOUANE FRANÇAISE	https://www.youtube.com/channel/AKbi-v4SkikcfjjQsGw-rA
**AOf87a3S24IgoiqsuwxHnQ**	Thierry Benoit	https://www.youtube.com/channel/AOf87a3S24IgoiqsuwxHnQ
**APbIIdEtsGPeZX4oOeyR9g**	Première magazine	https://www.youtube.com/channel/APbIIdEtsGPeZX4oOeyR9g
**AWbpsb5YQKxE8F62TEVCww**	Salim Laïbi - سليم لعيبي Le Libre Penseur	https://www.youtube.com/channel/AWbpsb5YQKxE8F62TEVCww
**AcAnMF0OrCtUep3Y4M-ZPw**	HugoDécrypte	https://www.youtube.com/channel/AcAnMF0OrCtUep3Y4M-ZPw
**ActIgCztmeejZwSbral2Nw**	La Manif Pour Tous	https://www.youtube.com/channel/ActIgCztmeejZwSbral2Nw
**Ad_SBAv7z9cV0p1HcHKelg**	Ornella N'Orny Vaudron	https://www.youtube.com/channel/Ad_SBAv7z9cV0p1HcHKelg
**AekeEO-OZaPg_ZvISWf4Lw**	Laurent febvay En tournée	https://www.youtube.com/channel/AekeEO-OZaPg_ZvISWf4Lw
**AfBTPNonzl7kgKvSsIXV9A**	Dimitri Houbron	https://www.youtube.com/channel/AfBTPNonzl7kgKvSsIXV9A
**AgUV11t0UuSn0BOF0BlZ3w**	docteur alwest	https://www.youtube.com/channel/AgUV11t0UuSn0BOF0BlZ3w
**ApzB6X-04YRPq9SZ0WfEsg**	ministère des Solidarités et de la Santé	https://www.youtube.com/channel/ApzB6X-04YRPq9SZ0WfEsg
**AtFVqd8Bs_5V8OPE3shIcg**	Le Dauphiné Libéré	https://www.youtube.com/channel/AtFVqd8Bs_5V8OPE3shIcg

**AyKkx7rjXke8qSoUmAdrxw**	L'HEURE DE SE REVEILLER	https://www.youtube.com/channel/AyKkx7rjXke8qSoUmAdrxw
**AyL93zSY9buLMdSZKc50QA**	ClicAnimaux	https://www.youtube.com/channel/AyL93zSY9buLMdSZKc50QA
**Az-755tH3m8_BwaluzdJwQ**	Numerama	https://www.youtube.com/channel/Az-755tH3m8_BwaluzdJwQ
**B1rFTFynmUznorQwoYioew**	Raphael Gerard	https://www.youtube.com/channel/B1rFTFynmUznorQwoYioew
**B32y6dm508Hap4_Mn-ZnHQ**	Presse-citron	https://www.youtube.com/channel/B32y6dm508Hap4_Mn-ZnHQ
**BEexcqqP2KRf84Lb787_Zg**	Jean-François PORTARRIEU	https://www.youtube.com/channel/BEexcqqP2KRf84Lb787_Zg
**BIyR71Yvq7rWHSF2p-uH4Q**	Campagnol tvl	https://www.youtube.com/channel/BIyR71Yvq7rWHSF2p-uH4Q
**BOEy0ETYHd5gWQ2DayMv_g**	Kriss Papillon	https://www.youtube.com/channel/BOEy0ETYHd5gWQ2DayMv_g
**BV0UXKsSD3S2XQMD20QrtQ**	Ecole polytechnique	https://www.youtube.com/channel/BV0UXKsSD3S2XQMD20QrtQ
**BWD5UBb6Ybv2VcI57Gh60A**	jmichel2you	https://www.youtube.com/channel/BWD5UBb6Ybv2VcI57Gh60A
**BYoReK1vXF7WMbgT4KVgmQ**	ademe	https://www.youtube.com/channel/BYoReK1vXF7WMbgT4KVgmQ
**BZLchaV-vn4R-NoaSN1klg**	Revue éléments	https://www.youtube.com/channel/BZLchaV-vn4R-NoaSN1klg
**BclKo3wjVBwR3ceHcnrQ4Q**	AGEFI TV	https://www.youtube.com/channel/BclKo3wjVBwR3ceHcnrQ4Q
**BlHyzrLWvjyBaFx1fFVvqg**	DECIDEURSTV	https://www.youtube.com/channel/BlHyzrLWvjyBaFx1fFVvqg
**Bm6S_t8xz00JoSGbqD5Zaw**	Le Cercle Richelieu	https://www.youtube.com/channel/Bm6S_t8xz00JoSGbqD5Zaw
**BpSbjzYAb5suUAHD-IaHcQ**	Lê Văn Trường	https://www.youtube.com/channel/BpSbjzYAb5suUAHD-IaHcQ
**BpatvFjRRxFt03q8tNf7UA**	Mayotte la 1ère	https://www.youtube.com/channel/BpatvFjRRxFt03q8tNf7UA
**BvamfHje2OdDnoycTAgPQw**	Collectif Contre l'Islamophobie en France	https://www.youtube.com/channel/BvamfHje2OdDnoycTAgPQw
**Bx_2HkT0w8ESW58_NrB-1w**	piero san giorgio	https://www.youtube.com/channel/Bx_2HkT0w8ESW58_NrB-1w
**CBR4hxc0hMbWs0IfBljQMg**	France Télévisions	https://www.youtube.com/channel/CBR4hxc0hMbWs0IfBljQMg
**CCFCxkPHFfuAVs9zwFiI0Q**	JC2R Officiel 2	https://www.youtube.com/channel/CCFCxkPHFfuAVs9zwFiI0Q
**CCPCZNChQdGa9EkATeye4g**	FRANCE 24	https://www.youtube.com/channel/CCPCZNChQdGa9EkATeye4g
**CDz_XYeKWd0OIyjp95dqyQ**	Figaro Live	https://www.youtube.com/channel/CDz_XYeKWd0OIyjp95dqyQ
**CG5P2uLpwoGs8OAprJ77bg**	FalloutVideo & Actus	https://www.youtube.com/channel/CG5P2uLpwoGs8OAprJ77bg
**CJMIP4tqfnIGFeB1ZsgM0g**	blondewoman1	https://www.youtube.com/channel/CJMIP4tqfnIGFeB1ZsgM0g
**CKVNfcXWvHNpKJZZlgQqAw**	Monor	https://www.youtube.com/channel/CKVNfcXWvHNpKJZZlgQqAw
**CM-fZ_k1pMrIC7fet83LFg**	Albane Gaillot	https://www.youtube.com/channel/CM-fZ_k1pMrIC7fet83LFg
**CNW4Qbv3N0oEB2oVu2BUtw**	francetv slash / enquêtes	https://www.youtube.com/channel/CNW4Qbv3N0oEB2oVu2BUtw
**CNyXbmCmmEJleukYgD1yMA**	Les Internettes	https://www.youtube.com/channel/CNyXbmCmmEJleukYgD1yMA
**CTuFofVWD9uORhQqgn-d7Q**	Ina Société	https://www.youtube.com/channel/CTuFofVWD9uORhQqgn-d7Q
**CUyfAgbTAsph3ZGhNSi70Q**	Les Choses au Claire	https://www.youtube.com/channel/CUyfAgbTAsph3ZGhNSi70Q
**CW216og7Av8_2cq25GrYJQ**	L214medias	https://www.youtube.com/channel/CW216og7Av8_2cq25GrYJQ
**CaFw8qbWdrdU6MYYGYwH2w**	Thomas Gassilloud	https://www.youtube.com/channel/CaFw8qbWdrdU6MYYGYwH2w
**CauEjHzPXxGer8af3u4x5g**	Envoyé Spécial	https://www.youtube.com/channel/CauEjHzPXxGer8af3u4x5g
**CcXckpueHQGu6s8OzaKEYg**	Xavier Paluszkiewicz, Député de Meurthe-et-Moselle	https://www.youtube.com/channel/CcXckpueHQGu6s8OzaKEYg
**CuySJIvnUg7Zrbcvb4zlgg**	La Quotidienne	https://www.youtube.com/channel/CuySJIvnUg7Zrbcvb4zlgg
**CzXLEXV-m8kUP5h8dRu8Yg**	lesnonalignes	https://www.youtube.com/channel/CzXLEXV-m8kUP5h8dRu8Yg
**D25QI36ly-8hWm9OPOz7aw**	20MinutesFR	https://www.youtube.com/channel/D25QI36ly-8hWm9OPOz7aw
**D3eoE_Kv1K3aUUFESPMTUg**	Le Croissant de lumière	https://www.youtube.com/channel/D3eoE_Kv1K3aUUFESPMTUg
**D3zqB9TdKZVnKAk8rdaq-g**	Frederic Delavier	https://www.youtube.com/channel/D3zqB9TdKZVnKAk8rdaq-g
**D5WTHI6dkvGshFGiUZudSg**	Bruno Lallement	https://www.youtube.com/channel/D5WTHI6dkvGshFGiUZudSg
**D6yZkXQ5TWfxgXiZjHiZag**	Actu Fr	https://www.youtube.com/channel/D6yZkXQ5TWfxgXiZjHiZag
**DHYLk7Ou2OkHSNsO6Cy3JA**	Ville de Clermont-Ferrand	https://www.youtube.com/channel/DHYLk7Ou2OkHSNsO6Cy3JA
**DKcvNGkX-1QxNoBt_Ac-zA**	Alexis Corbière	https://www.youtube.com/channel/DKcvNGkX-1QxNoBt_Ac-zA
**DMgDbI47fpx66wrhnUP3vg**	Michel Larive	https://www.youtube.com/channel/DMgDbI47fpx66wrhnUP3vg
**DZ18dPs6GJKFF80mKy8txg**	Caméra Gilets Jaunes	https://www.youtube.com/channel/DZ18dPs6GJKFF80mKy8txg
**DZSum0LrBcCyxC7T28l9wQ**	Lopez Frédéric	https://www.youtube.com/channel/DZSum0LrBcCyxC7T28l9wQ
**DZTep8HCLZGEaw7_I1cVCw**	Tendance Ouest	https://www.youtube.com/channel/DZTep8HCLZGEaw7_I1cVCw
**DbMQHClDaiGpRP2UEjCMog**	TV Replay	https://www.youtube.com/channel/DbMQHClDaiGpRP2UEjCMog
**DeQv6W8Obq_KfEmwVSl7JA**	AGORA	https://www.youtube.com/channel/DeQv6W8Obq_KfEmwVSl7JA
**DpFyuCQi-cD_2dwD9ITUEg**	Almosni Samuel	https://www.youtube.com/channel/DpFyuCQi-cD_2dwD9ITUEg
**E1OLr08s3BkrYpxz5FXY0Q**	Jean Francois Mbaye	https://www.youtube.com/channel/E1OLr08s3BkrYpxz5FXY0Q
**EG4q53oEgb7ZoAGrd7QeCA**	Assimil TV	https://www.youtube.com/channel/EG4q53oEgb7ZoAGrd7QeCA
**ENLWVOaouYne7G3fWV6jAg**	Olivier Dassault	https://www.youtube.com/channel/ENLWVOaouYne7G3fWV6jAg

**ENv8pH4LkzvuSV_qHIcslg**	C'est pas sorcier	https://www.youtube.com/channel/ENv8pH4LkzvuSV_qHIcslg
**EP3Okp7_QaCqe4yepB50Xg**	Thomas Ferrier	https://www.youtube.com/channel/EP3Okp7_QaCqe4yepB50Xg
**ESTwDXpoMgiYBHipMdKTkQ**	Sud Radio	https://www.youtube.com/channel/ESTwDXpoMgiYBHipMdKTkQ
**EWqsDR8cjoxxHAS7R8R9UQ**	La chaîne qui pique !	https://www.youtube.com/channel/EWqsDR8cjoxxHAS7R8R9UQ
**EYPFwoqCeTxgfwWmeC3Ung**	Alliance royale Le parti royaliste	https://www.youtube.com/channel/EYPFwoqCeTxgfwWmeC3Ung
**EZcV1ZuGsN-l4pkVyRwdtg**	Bernard Bordas	https://www.youtube.com/channel/EZcV1ZuGsN-l4pkVyRwdtg
**EgDWj0HxjOuc_rYHLtJARg**	Abel Chemoul	https://www.youtube.com/channel/EgDWj0HxjOuc_rYHLtJARg
**EpcAtDkyYJht97HsqUHbCw**	Reporter Youtube	https://www.youtube.com/channel/EpcAtDkyYJht97HsqUHbCw
**EqXcoGLgXYeb6vESoLkxUw**	AFAF agroforesterie	https://www.youtube.com/channel/EqXcoGLgXYeb6vESoLkxUw
**EqwfpB34mhHaAx-WE1Tqlg**	Bastien Lachaud, député insoumis	https://www.youtube.com/channel/EqwfpB34mhHaAx-WE1Tqlg
**Etsg2WH8HssrifbUhVQeRA**	KELCI	https://www.youtube.com/channel/Etsg2WH8HssrifbUhVQeRA
**F5XmjNN3FzNPn_VR1xTr8Q**	Teddyboy RSA	https://www.youtube.com/channel/F5XmjNN3FzNPn_VR1xTr8Q
**F6JBDGCxGtHABMxWlPRsdg**	Wéo, la télé Hauts-de-France	https://www.youtube.com/channel/F6JBDGCxGtHABMxWlPRsdg
**FAFYSa9BhHmH5zv2n6UzFQ**	Christian Mrasilevici	https://www.youtube.com/channel/FAFYSa9BhHmH5zv2n6UzFQ
**FBo7nOUvkL7icgcWyl3gNw**	Région Auvergne-Rhône-Alpes	https://www.youtube.com/channel/FBo7nOUvkL7icgcWyl3gNw
**FC3nJ8GXl5yONe3tB_vhsw**	France 3 Hauts-de-France	https://www.youtube.com/channel/FC3nJ8GXl5yONe3tB_vhsw
**FJEbkWsS1v7xkCuD786Iiw**	Pr. Henri Joyeux	https://www.youtube.com/channel/FJEbkWsS1v7xkCuD786Iiw
**FPTO55xxHqFqkzRZHu4kcw**	Virginie Vota	https://www.youtube.com/channel/FPTO55xxHqFqkzRZHu4kcw
**FZbM3WW2Tpt0diM9C_9LPQ**	deputessocialistesEU	https://www.youtube.com/channel/FZbM3WW2Tpt0diM9C_9LPQ
**FaPzuoXcACu7jplePDfXmA**	IHU Méditerranée-Infection	https://www.youtube.com/channel/FaPzuoXcACu7jplePDfXmA
**FcKiUrYezu0tEJfb86ixZQ**	Pagans TV	https://www.youtube.com/channel/FcKiUrYezu0tEJfb86ixZQ
**FdEMYmiimq2uKmOMaBfPLg**	Ina Histoire	https://www.youtube.com/channel/FdEMYmiimq2uKmOMaBfPLg
**Fe-p93CVJ13v3GM0hIzYNg**	Canard PC	https://www.youtube.com/channel/Fe-p93CVJ13v3GM0hIzYNg
**FgAEHHtZ67NvYI051jYfwQ**	Yahia GOUASMI	https://www.youtube.com/channel/FgAEHHtZ67NvYI051jYfwQ
**FiFXHHYfmxR6Fa_g6f4P1g**	Greenpeace France	https://www.youtube.com/channel/FiFXHHYfmxR6Fa_g6f4P1g
**FkAfcjj11xKklaMSngbXjQ**	Viking Eco	https://www.youtube.com/channel/FkAfcjj11xKklaMSngbXjQ
**FnwF4Nf0rbHHqJ_oe_kTLw**	NOP	https://www.youtube.com/channel/FnwF4Nf0rbHHqJ_oe_kTLw
**FqGa9uitcB-fWyNZK2xImw**	Emmanuel Macron	https://www.youtube.com/channel/FqGa9uitcB-fWyNZK2xImw
**FrDDP81MX_QfOHrRZOgD4g**	L214 éthique et animaux	https://www.youtube.com/channel/FrDDP81MX_QfOHrRZOgD4g
**G0PZ16zd6GMV7cTVY4IQrA**	John Doggett	https://www.youtube.com/channel/G0PZ16zd6GMV7cTVY4IQrA
**G6Zpr_CnDxASjeHyBHtQMw**	Pierre Jovanovic - La Revue de Presse	https://www.youtube.com/channel/G6Zpr_CnDxASjeHyBHtQMw
**GAR1p-zHqYAKo2Y1EtMnrg**	Santé publique France	https://www.youtube.com/channel/GAR1p-zHqYAKo2Y1EtMnrg
**GEU07FoKKAqLSg4PpMj0kQ**	Publications Agora	https://www.youtube.com/channel/GEU07FoKKAqLSg4PpMj0kQ
**GEojLXQO-jbQ6a0DD1HG3Q**	Le Yéti	https://www.youtube.com/channel/GEojLXQO-jbQ6a0DD1HG3Q
**GJ43pRU4fRbL1ucLRavVGQ**	l'atelier de Gépéto Mouvement espérance	https://www.youtube.com/channel/GJ43pRU4fRbL1ucLRavVGQ
**GZW879__5Z3xBtw2FSsEeg**	Sea Shepherd France - Officiel	https://www.youtube.com/channel/GZW879__5Z3xBtw2FSsEeg
**GeFgMJfWclTWuPw8Ok5FUQ**	horizon-gull	https://www.youtube.com/channel/GeFgMJfWclTWuPw8Ok5FUQ
**GhyQZ6zPbEsEAmA_IswvrA**	Légion étrangère	https://www.youtube.com/channel/GhyQZ6zPbEsEAmA_IswvrA
**Gl2QLR344ry4Y20RV9dM3g**	FEUILLAGE	https://www.youtube.com/channel/Gl2QLR344ry4Y20RV9dM3g
**Gp0ZbEM4j6RA4jufMgG3FA**	Guadeloupe la 1ère	https://www.youtube.com/channel/Gp0ZbEM4j6RA4jufMgG3FA
**GtQeqbi89Y2hJRoi3zBgRQ**	Jean-Charles Colas-Roy	https://www.youtube.com/channel/GtQeqbi89Y2hJRoi3zBgRQ
**GvjUWz3mGV9cssraATuEsw**	Radio Nova	https://www.youtube.com/channel/GvjUWz3mGV9cssraATuEsw
**GxEJXuCo8lXti1A3akxyjg**	Télé Matin	https://www.youtube.com/channel/GxEJXuCo8lXti1A3akxyjg
**GxU0geTBrKh4VpbXFGF_jg**	Ça Zap !	https://www.youtube.com/channel/GxU0geTBrKh4VpbXFGF_jg
**H0XvUpYcxn4V0iZGnZXMnQ**	Lama Faché	https://www.youtube.com/channel/H0XvUpYcxn4V0iZGnZXMnQ
**H6BP1Vz9hi2MaPjHyIZ8xg**	Laïcité République	https://www.youtube.com/channel/H6BP1Vz9hi2MaPjHyIZ8xg
**H6rAZUDfVIoVSJjm3vlcnw**	L'Esprit Sorcier Officiel	https://www.youtube.com/channel/H6rAZUDfVIoVSJjm3vlcnw
**HGFbA0KWBgf6gMbyUCZeCQ**	LCP Assemblée nationale	https://www.youtube.com/channel/HGFbA0KWBgf6gMbyUCZeCQ
**HGMBrXUzClgjEzBMei-Jdw**	Le Dessous des Cartes - ARTE	https://www.youtube.com/channel/HGMBrXUzClgjEzBMei-Jdw
**HL_3cEz7pqDx4gpnzAdq_g**	Jean-Luc Lagleize - Député de la Haute-Garonne	https://www.youtube.com/channel/HL_3cEz7pqDx4gpnzAdq_g
**HMYJ0izNnV9R21D43ZVPhw**	Lyon Capitale Live	https://www.youtube.com/channel/HMYJ0izNnV9R21D43ZVPhw
**HOtlkL8XEHbL6KHIHYRGSw**	Franck Lepage	https://www.youtube.com/channel/HOtlkL8XEHbL6KHIHYRGSw
**HQ_RquyROcW-5RefegCPJQ**	FDMTV - France Diversité Média	https://www.youtube.com/channel/HQ_RquyROcW-5RefegCPJQ
**HQda5vLxrH0Ff0I0kMq4zw**	Konbini	https://www.youtube.com/channel/HQda5vLxrH0Ff0I0kMq4zw

**HRPg4LxfQGag83gQWfXYAQ**	Dieudonné Officiel	https://www.youtube.com/channel/HRPg4LxfQGag83gQWfXYAQ
**HSorAIx34bQ76TFwNyJrQw**	Younes Yanis	https://www.youtube.com/channel/HSorAIx34bQ76TFwNyJrQw
**HTAY50XLBOOoIrH-_vv3NA**	Joharno	https://www.youtube.com/channel/HTAY50XLBOOoIrH-_vv3NA
**HTEGIA9QrWAOw6cCeHX0FQ**	La France insoumise - Groupe parlementaire	https://www.youtube.com/channel/HTEGIA9QrWAOw6cCeHX0FQ
**HTPWexSZGfyM5Ph3al0SDw**	France 3 Basse-Normandie	https://www.youtube.com/channel/HTPWexSZGfyM5Ph3al0SDw
**HVF0XnnAptKFE9xskvXblA**	La République des Pyrénées	https://www.youtube.com/channel/HVF0XnnAptKFE9xskvXblA
**HZJkDU1pakkDygpUkuj6tA**	Alain Bruneel	https://www.youtube.com/channel/HZJkDU1pakkDygpUkuj6tA
**HdnhpYaJHKz0tBEf3Z1KAw**	Franck Brusset	https://www.youtube.com/channel/HdnhpYaJHKz0tBEf3Z1KAw
**Hg3-y8Yf-a3VG0YPSlTMkA**	Anthony Joubert	https://www.youtube.com/channel/Hg3-y8Yf-a3VG0YPSlTMkA
**Hj3iul0TIdY1jbssgIuUeQ**	koi de neuf? Buzz	https://www.youtube.com/channel/Hj3iul0TIdY1jbssgIuUeQ
**Hr3CLFqgzkLqS1cQBwY4eA**	Journal de la Haute-Marne	https://www.youtube.com/channel/Hr3CLFqgzkLqS1cQBwY4eA
**Hy8kwF57Kh-YflbJ0WsMoA**	Lumière sur Gaia	https://www.youtube.com/channel/Hy8kwF57Kh-YflbJ0WsMoA
**I-4eGs7LgjGCdGUbQy1w4A**	Ajenwy 92	https://www.youtube.com/channel/I-4eGs7LgjGCdGUbQy1w4A
**I876FbErhdaxIA9kLaQJ1A**	louisette lavraie	https://www.youtube.com/channel/I876FbErhdaxIA9kLaQJ1A
**I8QAqi_0wDxHiHFcbLyqJQ**	lachainedevv	https://www.youtube.com/channel/I8QAqi_0wDxHiHFcbLyqJQ
**ID-NICViVhXHTzTDTVXE0w**	Auto Plus Magazine	https://www.youtube.com/channel/ID-NICViVhXHTzTDTVXE0w
**IJffkItaprUTFmDNOMXYxA**	SANGLIER JAUNE	https://www.youtube.com/channel/IJffkItaprUTFmDNOMXYxA
**IMGfEAERXjmWwQeg15BFsg**	Europe 1	https://www.youtube.com/channel/IMGfEAERXjmWwQeg15BFsg
**IO0R7N49K14bBvWIEXPYFg**	iReMMO	https://www.youtube.com/channel/IO0R7N49K14bBvWIEXPYFg
**IQGSp79vVch0vO3Efqif_w**	FRANÇOIS RUFFIN	https://www.youtube.com/channel/IQGSp79vVch0vO3Efqif_w
**IV-NTLRUBzAY404qiQ1OYw**	Pôle emploi	https://www.youtube.com/channel/IV-NTLRUBzAY404qiQ1OYw
**IWw8PPP5OPNkDCLBrG9zVA**	NTDFrench	https://www.youtube.com/channel/IWw8PPP5OPNkDCLBrG9zVA
**IgLkuNUxjRRAFgdCnIpcjw**	lemainelibre	https://www.youtube.com/channel/IgLkuNUxjRRAFgdCnIpcjw
**IkYvjt9MFLY0gmYlpt-Z2w**	On n'demande qu'à en rire	https://www.youtube.com/channel/IkYvjt9MFLY0gmYlpt-Z2w
**IlG4CC0a_XaJVqmGteFFfg**	Sophie Taillé-Polian	https://www.youtube.com/channel/IlG4CC0a_XaJVqmGteFFfg
**Iu1KhZAvIiujTIgNAz22sQ**	Ridicule TV	https://www.youtube.com/channel/Iu1KhZAvIiujTIgNAz22sQ
**IwyAXIOfX0u5tRwxsQeyzQ**	UnivJeanMoulin Lyon3	https://www.youtube.com/channel/IwyAXIOfX0u5tRwxsQeyzQ
**IxTyNfetgZ9O0jhEDemA5g**	jean bricmont	https://www.youtube.com/channel/IxTyNfetgZ9O0jhEDemA5g
**J24mTwxSj-xyOvHXXNkgRw**	AIDES	https://www.youtube.com/channel/J24mTwxSj-xyOvHXXNkgRw
**J2KjVGTtD0Cb6UX4Z5fW4w**	Secretariat Elisabeth Toutut-Picard	https://www.youtube.com/channel/J2KjVGTtD0Cb6UX4Z5fW4w
**J9kIKgFuAB23GkEko1hHDg**	Mycéliums	https://www.youtube.com/channel/J9kIKgFuAB23GkEko1hHDg
**J9tHJbCa8ZoMbapfJLy0ZQ**	imineo Documentaires	https://www.youtube.com/channel/J9tHJbCa8ZoMbapfJLy0ZQ
**JBmrvimOZNMCKoYr2k5_Yw**	AFD - Agence Française de Développement	https://www.youtube.com/channel/JBmrvimOZNMCKoYr2k5_Yw
**JC-W0PpnKHos9rHSJOoAJA**	Komrad	https://www.youtube.com/channel/JC-W0PpnKHos9rHSJOoAJA
**JIl08OJstq_9kgI9wf_xjg**	Groland Le Zapoï	https://www.youtube.com/channel/JIl08OJstq_9kgI9wf_xjg
**JMqaBnrPa0mTO45CFHeAaA**	Olga Givernet	https://www.youtube.com/channel/JMqaBnrPa0mTO45CFHeAaA
**JVYmreAAbWCHj6i4GykjAA**	UDI Agir et Indépendants	https://www.youtube.com/channel/JVYmreAAbWCHj6i4GykjAA
**JeiOCUqrEbMqvqjuYVSihQ**	BNP Paribas	https://www.youtube.com/channel/JeiOCUqrEbMqvqjuYVSihQ
**JeinKLg1dOGIOvS9m07vcg**	Université PSL	https://www.youtube.com/channel/JeinKLg1dOGIOvS9m07vcg
**JgNMdfAExlU7MwHlET6XCw**	France 3 Paris Ile-de-France	https://www.youtube.com/channel/JgNMdfAExlU7MwHlET6XCw
**JgY8TLEJ7kDK9NlTlwgwVg**	La Mite dans la Caverne	https://www.youtube.com/channel/JgY8TLEJ7kDK9NlTlwgwVg
**JhLDoms_DyjavCFdNCyANA**	D-crypte	https://www.youtube.com/channel/JhLDoms_DyjavCFdNCyANA
**Jk4JCxqck6vPxkcNiQY82w**	NFCA MEDIA	https://www.youtube.com/channel/Jk4JCxqck6vPxkcNiQY82w
**JldRgT_D7Am-ErRHQZ90uw**	France Inter	https://www.youtube.com/channel/JldRgT_D7Am-ErRHQZ90uw
**JrKxCxGvDPnFeMrOBm9WDA**	Elodie Jacquier-Laforge	https://www.youtube.com/channel/JrKxCxGvDPnFeMrOBm9WDA
**JsZHPR1jqKu-soDmKNMBFg**	TV5MONDE	https://www.youtube.com/channel/JsZHPR1jqKu-soDmKNMBFg
**JvlI4LdBGk1tkALBKUJjkg**	CDjamelito	https://www.youtube.com/channel/JvlI4LdBGk1tkALBKUJjkg
**Jw8np695wqWOaKVhFjkRyg**	La République En Marche !	https://www.youtube.com/channel/Jw8np695wqWOaKVhFjkRyg
**K6mlWEjd5-wK1aOqL2zKQQ**	Point de vue social #PDVS	https://www.youtube.com/channel/K6mlWEjd5-wK1aOqL2zKQQ
**KDIO0PuLi4_wxfyajE7V5A**	Frédéric Descrozaille	https://www.youtube.com/channel/KDIO0PuLi4_wxfyajE7V5A
**KHKSD-yanY2ZwwU_4Tgf0w**	La France insoumise	https://www.youtube.com/channel/KHKSD-yanY2ZwwU_4Tgf0w
**KQKLYeuCqcQbLV4-1chMjQ**	esobook	https://www.youtube.com/channel/KQKLYeuCqcQbLV4-1chMjQ
**KSr1nlWwCQzYbW_wUU8A3w**	Sott Media	https://www.youtube.com/channel/KSr1nlWwCQzYbW_wUU8A3w
**KbKn37cnCaFIYdkLp8AjMQ**	VirgOasis	https://www.youtube.com/channel/KbKn37cnCaFIYdkLp8AjMQ

**KhWMZRG_CN-S8_9UmC6PoA**	Les news de la famille	https://www.youtube.com/channel/KhWMZRG_CN-S8_9UmC6PoA
**KnlPeqsg9c7pZBYfJn5UTw**	Kernews	https://www.youtube.com/channel/KnlPeqsg9c7pZBYfJn5UTw
**KsOdXVAy9GomPMXGPX4tmg**	Outremers 360	https://www.youtube.com/channel/KsOdXVAy9GomPMXGPX4tmg
**KzEtXZNnk-rs2rMgirOCrw**	Thomas Mesnier	https://www.youtube.com/channel/KzEtXZNnk-rs2rMgirOCrw
**L5mpqESV-ntdIB7HYdoRzg**	CIA Chaine info d 'une Alien	https://www.youtube.com/channel/L5mpqESV-ntdIB7HYdoRzg
**L6QQLpH4T5oyiwVuDn8juQ**	Bilger Philippe	https://www.youtube.com/channel/L6QQLpH4T5oyiwVuDn8juQ
**L7CT3cjpvJNmzyZ0FNIayw**	Survivaliste Bushcrafter	https://www.youtube.com/channel/L7CT3cjpvJNmzyZ0FNIayw
**L95UhlTaJ5YNJczY5I-X5A**	Les Goguettes	https://www.youtube.com/channel/L95UhlTaJ5YNJczY5I-X5A
**LDRl9dB6Q_7ta0U2TpcCUg**	Cercles Nationalistes Français	https://www.youtube.com/channel/LDRl9dB6Q_7ta0U2TpcCUg
**LHlsGMNqgfZbVFjRoy8_iA**	Le Poste	https://www.youtube.com/channel/LHlsGMNqgfZbVFjRoy8_iA
**LIK_dUpO9ffS5mAe_ouiSA**	Where the Claim is	https://www.youtube.com/channel/LIK_dUpO9ffS5mAe_ouiSA
**LIrMXjsNxhvVQR-YybDGfQ**	Jasper Mader	https://www.youtube.com/channel/LIrMXjsNxhvVQR-YybDGfQ
**LJ9WtC9PoiwvVzpzyTcrrA**	Droitards Méchants	https://www.youtube.com/channel/LJ9WtC9PoiwvVzpzyTcrrA
**LXCYpbD-9TnoX_cXSbVLjw**	Elsa FAUCILLON, Députée	https://www.youtube.com/channel/LXCYpbD-9TnoX_cXSbVLjw
**L_cZf5sHKQHMRIEax5o3sg**	ARTE Découverte	https://www.youtube.com/channel/L_cZf5sHKQHMRIEax5o3sg
**M0FoZDcJdoq_ekCa99rcaA**	IL EST MINUIT	https://www.youtube.com/channel/M0FoZDcJdoq_ekCa99rcaA
**M1dPrcar1pIz0EwfXDHzBQ**	Le Maquis à Paris	https://www.youtube.com/channel/M1dPrcar1pIz0EwfXDHzBQ
**M4EpJC1Jrqi--jJ3YfKSKQ**	Odon Lafontaine	https://www.youtube.com/channel/M4EpJC1Jrqi--jJ3YfKSKQ
**M5WOJ5hxx6Ul1ks3BpkjNw**	N E W s H ALL iens 365	https://www.youtube.com/channel/M5WOJ5hxx6Ul1ks3BpkjNw
**M6fXSZL41Z6LKL7Jic8iag**	DarnaTelevision	https://www.youtube.com/channel/M6fXSZL41Z6LKL7Jic8iag
**MAT1VA7o6bD6qnpBBZ_New**	Là-bas si j'y suis	https://www.youtube.com/channel/MAT1VA7o6bD6qnpBBZ_New
**MAbRPmQNgRnVCTSC1FTheg**	Stéphane Mazars	https://www.youtube.com/channel/MAbRPmQNgRnVCTSC1FTheg
**MDPDRDGBUKE0Fg4BE8jkdA**	Wendy Phele	https://www.youtube.com/channel/MDPDRDGBUKE0Fg4BE8jkdA
**MFcMhePnH4onVHt2-ItPZw**	Hygiène Mentale	https://www.youtube.com/channel/MFcMhePnH4onVHt2-ItPZw
**MOcYAHXBDTV41He19dKaPA**	Faculté des Lettres de Sorbonne Université	https://www.youtube.com/channel/MOcYAHXBDTV41He19dKaPA
**MOrzCo7Jdp6qqEX24CXuog**	Juan Branco	https://www.youtube.com/channel/MOrzCo7Jdp6qqEX24CXuog
**MPIMCKpq5TRd92Jwj2D7_Q**	France 3 Bourgogne-Franche-Comté	https://www.youtube.com/channel/MPIMCKpq5TRd92Jwj2D7_Q
**MPi5y3uwrWHd-Q73A0XbFQ**	did B	https://www.youtube.com/channel/MPi5y3uwrWHd-Q73A0XbFQ
**MPnD8fhwPMk_9XsYfc96JQ**	France 3 Provence-Alpes Côte d'Azur	https://www.youtube.com/channel/MPnD8fhwPMk_9XsYfc96JQ
**MQSwUNSnMOP4IRysfeg8EA**	Simon Puech	https://www.youtube.com/channel/MQSwUNSnMOP4IRysfeg8EA
**MRJqoSRIaakAJUJK104Z8Q**	Touche pas à mon poste !	https://www.youtube.com/channel/MRJqoSRIaakAJUJK104Z8Q
**MaO2u7ZMuyPVzj5h_CDl3A**	Micka Vlog du quotidien	https://www.youtube.com/channel/MaO2u7ZMuyPVzj5h_CDl3A
**MbAn5levW-QC-cARoLs0tg**	Rouen Dans La Rue	https://www.youtube.com/channel/MbAn5levW-QC-cARoLs0tg
**MdHCyu-W36i8Z4RWnIPWEw**	Le Courrier de Russie	https://www.youtube.com/channel/MdHCyu-W36i8Z4RWnIPWEw
**MkxZzrQwdyIs6EBbreyH1g**	The SeaCleaners	https://www.youtube.com/channel/MkxZzrQwdyIs6EBbreyH1g
**MrkGXHiTg644GoZ2YKNDZA**	Christophe Naegelen	https://www.youtube.com/channel/MrkGXHiTg644GoZ2YKNDZA
**N-2-BfDdHwLo1zpERLV5ZA**	D1ST1 OFFICIEL RAP	https://www.youtube.com/channel/N-2-BfDdHwLo1zpERLV5ZA
**N-ihxA9gI8_-RaEMCsPCIA**	AdcaZz	https://www.youtube.com/channel/N-ihxA9gI8_-RaEMCsPCIA
**N-sa7hXN_-0SvnZ3QFgErA**	Télé Star	https://www.youtube.com/channel/N-sa7hXN_-0SvnZ3QFgErA
**N-z2zxKXB1I8vvGWzyK1qg**	Scoop Volant	https://www.youtube.com/channel/N-z2zxKXB1I8vvGWzyK1qg
**N1ncvibKEDRYweziBjxm0A**	Gilets Jaunes 24/24 7/7 Relai Info	https://www.youtube.com/channel/N1ncvibKEDRYweziBjxm0A
**N5EzY5KpjbpB2468jZlN6A**	Université Sorbonne Paris Nord	https://www.youtube.com/channel/N5EzY5KpjbpB2468jZlN6A
**NBD4uZG6nWH2MMdgGESisw**	Ina Talk Shows	https://www.youtube.com/channel/NBD4uZG6nWH2MMdgGESisw
**NEWnUoWy0h8_6Fyt_59N1Q**	M - Gigantoraptor	https://www.youtube.com/channel/NEWnUoWy0h8_6Fyt_59N1Q
**NM-UkIP1BL5jv9ZrN5JMCA**	Caljbeut	https://www.youtube.com/channel/NM-UkIP1BL5jv9ZrN5JMCA
**NQUqp5ZcmOXNHzZYHRUnZ(**	La Luciole Mélenchantée	https://www.youtube.com/channel/NQUqp5ZcmOXNHzZYHRUnZ(
**NR2kAJlNnffO-jOLL5DnrQ**	Merri	https://www.youtube.com/channel/NR2kAJlNnffO-jOLL5DnrQ
**NV__KeK9vWDEzVOTxMTdhw**	Audrey Dufeu Schubert	https://www.youtube.com/channel/NV__KeK9vWDEzVOTxMTdhw
**NWajg-3XAYPEJFBXavcHRQ**	Sidaction	https://www.youtube.com/channel/NWajg-3XAYPEJFBXavcHRQ
**NWs0QTTHm7yiPMwl0aynsg**	code- Reinho	https://www.youtube.com/channel/NWs0QTTHm7yiPMwl0aynsg
**NdLt_AwDH3C_0Wu3Rcvw0g**	Topissime	https://www.youtube.com/channel/NdLt_AwDH3C_0Wu3Rcvw0g
**NdrMeOzE3P_6d8vD9htSEQ**	Le Nouveau Detective	https://www.youtube.com/channel/NdrMeOzE3P_6d8vD9htSEQ
**NjKSl5Oj1cgLeQtSRi5_6w**	Laurent Martinez	https://www.youtube.com/channel/NjKSl5Oj1cgLeQtSRi5_6w
**NovJemYKcdKt7PDdptJZfQ**	Jean-Marc Jancovici	https://www.youtube.com/channel/NovJemYKcdKt7PDdptJZfQ

**NpTz2M9uelipe_2UMiorQg**	Le Suisse	https://www.youtube.com/channel/NpTz2M9uelipe_2UMiorQg
**Npjt1HR25fpXmb-H28yajQ**	Arcana les Mystères du Monde	https://www.youtube.com/channel/Npjt1HR25fpXmb-H28yajQ
**NrMroWIXHBtuPOFwFBk65A**	UnivNantes	https://www.youtube.com/channel/NrMroWIXHBtuPOFwFBk65A
**NsyUg9n7TB365g4n13UOIg**	Le Vent Se Lève	https://www.youtube.com/channel/NsyUg9n7TB365g4n13UOIg
**O3yT7Q8jkQWE5TOxyx3DDQ**	ActuEntreprise	https://www.youtube.com/channel/O3yT7Q8jkQWE5TOxyx3DDQ
**O6K_kkdP-InSCiO3tPx7WA**	franceinfo	https://www.youtube.com/channel/O6K_kkdP-InSCiO3tPx7WA
**OBMbaiQeFI4PNDa18IQkLg**	Ugo Bernalicis	https://www.youtube.com/channel/OBMbaiQeFI4PNDa18IQkLg
**ODxvcNsW3aadQfVaUvpNYQ**	Pierre-Yves Bournazel	https://www.youtube.com/channel/ODxvcNsW3aadQfVaUvpNYQ
**OHORPRz79_zTAnZoOPLa4w**	France 24/7	https://www.youtube.com/channel/OHORPRz79_zTAnZoOPLa4w
**ONgKdISRnSKEZjCHviTLKA**	Philippe PASCOT	https://www.youtube.com/channel/ONgKdISRnSKEZjCHviTLKA
**ORWYsrGnBrCr-7FUbYy88A**	ANTILLUMINATI KEMAR 2	https://www.youtube.com/channel/ORWYsrGnBrCr-7FUbYy88A
**OXda0FH-DGhk82fU_c6s1g**	Les Nouvelles de Sablé	https://www.youtube.com/channel/OXda0FH-DGhk82fU_c6s1g
**OazMlysfZvHFA1pESaDf8w**	Crashdebug.fr, l'actu sans détour	https://www.youtube.com/channel/OazMlysfZvHFA1pESaDf8w
**Ob-zF_IKhXusbXln-q-GTA**	Erick Bernard	https://www.youtube.com/channel/Ob-zF_IKhXusbXln-q-GTA
**Odb0mpyr2YfukyTg4SLIWA**	Jean-Paul Lecoq	https://www.youtube.com/channel/Odb0mpyr2YfukyTg4SLIWA
**OfgAzxl1HYovJXBl4yBVEg**	Politique France	https://www.youtube.com/channel/OfgAzxl1HYovJXBl4yBVEg
**OuNP9ryCFZeY1S6RushYQQ**	Xavier-Louis de Izarra	https://www.youtube.com/channel/OuNP9ryCFZeY1S6RushYQQ
**OyXNHeljy0zrq7eBm-L_Jw**	F3Picardie	https://www.youtube.com/channel/OyXNHeljy0zrq7eBm-L_Jw
**OzXdA8UnCXo4r1e_2Ey5mA**	Loïc Prud'homme député insoumis	https://www.youtube.com/channel/OzXdA8UnCXo4r1e_2Ey5mA
**P2m0GEtx97dYpUnuhk7CIg**	Les Inrockuptibles	https://www.youtube.com/channel/P2m0GEtx97dYpUnuhk7CIg
**P46_MXP_WG_auH88FnfS1A**	Nota Bene	https://www.youtube.com/channel/P46_MXP_WG_auH88FnfS1A
**PD_ARnv3_TGGnBxzLr9bWw**	Lettres It Be	https://www.youtube.com/channel/PD_ARnv3_TGGnBxzLr9bWw
**PaeEhnVIdn4T01gUphIltw**	Élysée	https://www.youtube.com/channel/PaeEhnVIdn4T01gUphIltw
**Pbb-pFUWdT2DCTBCkdWCIA**	Allons Enfants	https://www.youtube.com/channel/Pbb-pFUWdT2DCTBCkdWCIA
**PdYeGxogYygpaSy4I2atIQ**	wanted info	https://www.youtube.com/channel/PdYeGxogYygpaSy4I2atIQ
**PgePfG6Nm06L9QQA3-I_aA**	ER LILLE	https://www.youtube.com/channel/PgePfG6Nm06L9QQA3-I_aA
**PhACJPOrdHabeo5_xfFRdA**	Mx Cordélia	https://www.youtube.com/channel/PhACJPOrdHabeo5_xfFRdA
**Piye7__vJMKdBxebtERd7g**	Terra Bellum	https://www.youtube.com/channel/Piye7__vJMKdBxebtERd7g
**PjBZT0aJPgJ3e1wAftV6TQ**	Ina Culte	https://www.youtube.com/channel/PjBZT0aJPgJ3e1wAftV6TQ
**PkoA795xDha5eAUFjISlwA**	Penseur Sauvage	https://www.youtube.com/channel/PkoA795xDha5eAUFjISlwA
**PlxmWbGbwVM2baH8q7MK6w**	Ina.fr Officiel	https://www.youtube.com/channel/PlxmWbGbwVM2baH8q7MK6w
**PplJEsqnAH_8s8qvyI3BEw**	Complément d'enquête	https://www.youtube.com/channel/PplJEsqnAH_8s8qvyI3BEw
**PrvtBhnf1-tORMrklK25yQ**	Jean-Paul MATTEI	https://www.youtube.com/channel/PrvtBhnf1-tORMrklK25yQ
**PtB9ESuNQdbW2BcTqEpt5Q**	Chaîne Catholique d'Arnaud Dumouch	https://www.youtube.com/channel/PtB9ESuNQdbW2BcTqEpt5Q
**PtoD96ccH6TNad0-OAtLzA**	Journal La Montagne	https://www.youtube.com/channel/PtoD96ccH6TNad0-OAtLzA
**Q1vo0N_ogXpQJ9AF3F5OZw**	La Presse de la Manche	https://www.youtube.com/channel/Q1vo0N_ogXpQJ9AF3F5OZw
**Q33jBrna4A874O5clR_DNQ**	Crayon jaune	https://www.youtube.com/channel/Q33jBrna4A874O5clR_DNQ
**QILGAU4t6MyO2zgnMGflgQ**	CONTRE-PROPAGANDE	https://www.youtube.com/channel/QILGAU4t6MyO2zgnMGflgQ
**QKmfKnJ9ZpNLGxjt0RiMJA**	Mouv'	https://www.youtube.com/channel/QKmfKnJ9ZpNLGxjt0RiMJA
**QOT2PpSDGo8pVOXJrJ721g**	TANRESI CENSUREEparDesFDP	https://www.youtube.com/channel/QOT2PpSDGo8pVOXJrJ721g
**QWkfJ5xVnprobdpKAGZ1fQ**	Le CERA	https://www.youtube.com/channel/QWkfJ5xVnprobdpKAGZ1fQ
**Qa2azr9n4y1JOOtKxoU-HQ**	RASTA PRESIDENT	https://www.youtube.com/channel/Qa2azr9n4y1JOOtKxoU-HQ
**QbDPKC0b_Fh4EKMeB6hWYQ**	Vincent Descoeur	https://www.youtube.com/channel/QbDPKC0b_Fh4EKMeB6hWYQ
**Qd5LYcykaFeZtjDaP3bNSA**	Le Point	https://www.youtube.com/channel/Qd5LYcykaFeZtjDaP3bNSA
**QeMSCfyA20wKktdsId5Lug**	Barbara Bessot Ballot, députée de Haute-Saône	https://www.youtube.com/channel/QeMSCfyA20wKktdsId5Lug
**QfzgoCt72pQNpkvnHjNvog**	The Conversation	https://www.youtube.com/channel/QfzgoCt72pQNpkvnHjNvog
**QgWpmt02UtJkyO32HGUASQ**	Thinkerview	https://www.youtube.com/channel/QgWpmt02UtJkyO32HGUASQ
**QiLl4wFWHnGn4LSjfDPOSA**	bordcham	https://www.youtube.com/channel/QiLl4wFWHnGn4LSjfDPOSA
**Qs8MrktndWkXdqOj08ogrQ**	Topito	https://www.youtube.com/channel/Qs8MrktndWkXdqOj08ogrQ
**QvNFAufGf8mtN2_L8thysg**	Journal du Geek	https://www.youtube.com/channel/QvNFAufGf8mtN2_L8thysg
**R5V8BTvlkyYtw6T_9swNEw**	RMC Sport	https://www.youtube.com/channel/R5V8BTvlkyYtw6T_9swNEw
**R9raBqPQisip_qnuCaUL1w**	Lumni	https://www.youtube.com/channel/R9raBqPQisip_qnuCaUL1w
**RAbwEqGDnUBt_gPOkplGBA**	L'instant détox	https://www.youtube.com/channel/RAbwEqGDnUBt_gPOkplGBA
**RAdymUfW2fDk3WNz6Hhf1w**	Dada Bens L'Art est public EN MARGE !	https://www.youtube.com/channel/RAdymUfW2fDk3WNz6Hhf1w

**REhQcvc7n7zLWPzK--CopA**	Députés Socialistes et apparentés	https://www.youtube.com/channel/REhQcvc7n7zLWPzK--CopA
**RH3paNAfcnkV5fORI1e8DQ**	L'informateur	https://www.youtube.com/channel/RH3paNAfcnkV5fORI1e8DQ
**RPi4B-d-u3FXx0XJ_qXf6w**	Mouvement Colibris	https://www.youtube.com/channel/RPi4B-d-u3FXx0XJ_qXf6w
**RQK1RZvLtvF5n53_yfEL7Q**	Break News TV	https://www.youtube.com/channel/RQK1RZvLtvF5n53_yfEL7Q
**RWLMiSuj9h0TMuWDZx7pUQ**	LetudiantTV	https://www.youtube.com/channel/RWLMiSuj9h0TMuWDZx7pUQ
**RfwKmBnl_ojeA60VLkUCow**	Vendée - Le Département	https://www.youtube.com/channel/RfwKmBnl_ojeA60VLkUCow
**Rh9jpKOj-qUIKJCSkOwvsw**	Luc Carvounas	https://www.youtube.com/channel/Rh9jpKOj-qUIKJCSkOwvsw
**RiL96aAW9OXCN0SiOSz7sQ**	Typhanie Degois	https://www.youtube.com/channel/RiL96aAW9OXCN0SiOSz7sQ
**RiLlth5A0HyW8Zixp9Bltg**	l'est-éclair journal	https://www.youtube.com/channel/RiLlth5A0HyW8Zixp9Bltg
**RkfmXUdbwiS759q6MOiY9A**	Eric drouet	https://www.youtube.com/channel/RkfmXUdbwiS759q6MOiY9A
**Rs50RPatI9YNzcpmiDUuWA**	Rémy CelMar	https://www.youtube.com/channel/Rs50RPatI9YNzcpmiDUuWA
**Ry5TG3g-OJzRXRbOnC2wpw**	Onpassealacte!	https://www.youtube.com/channel/Ry5TG3g-OJzRXRbOnC2wpw
**S1EeBm6TeQmZXpdRaAzieg**	Djemadine	https://www.youtube.com/channel/S1EeBm6TeQmZXpdRaAzieg
**S1lRt6He-GQGhV7W6VeD8w**	Ministères de Bercy	https://www.youtube.com/channel/S1lRt6He-GQGhV7W6VeD8w
**SDA159JHm9eQR5z7-TFvug**	Samantha Cazebonne	https://www.youtube.com/channel/SDA159JHm9eQR5z7-TFvug
**SF27JlpvPSRnsQVgqm-Ohw**	Kopp Johnson	https://www.youtube.com/channel/SF27JlpvPSRnsQVgqm-Ohw
**SFY15spR_TTeqD8EWVnXgg**	Verdi	https://www.youtube.com/channel/SFY15spR_TTeqD8EWVnXgg
**SKdvgqdnj72_SLggp7BDTg**	Brut	https://www.youtube.com/channel/SKdvgqdnj72_SLggp7BDTg
**SO6T55RFXOSTSjerLa1omg**	C à dire	https://www.youtube.com/channel/SO6T55RFXOSTSjerLa1omg
**SOAMLQ7vgAG5TQjidnn9Zg**	Hauteurs UGA	https://www.youtube.com/channel/SOAMLQ7vgAG5TQjidnn9Zg
**STCfUfo3JCs1bggH8quUtA**	CLIC RIC	https://www.youtube.com/channel/STCfUfo3JCs1bggH8quUtA
**SULDz1yaHLVQWHpm4g_GHA**	monsieur bidouille	https://www.youtube.com/channel/SULDz1yaHLVQWHpm4g_GHA
**SXSGW5mPkZ_yejHuTH30iA**	Buon TAN	https://www.youtube.com/channel/SXSGW5mPkZ_yejHuTH30iA
**S_7tplUgzJG4DhA16re5Yg**	Balade Mentale	https://www.youtube.com/channel/S_7tplUgzJG4DhA16re5Yg
**Skhq5LmpaIrbvvwd1zfmCA**	jayslem jayslem 2	https://www.youtube.com/channel/Skhq5LmpaIrbvvwd1zfmCA
**SkwGlUSFoolH727C6OIwwA**	Stéphane Peu	https://www.youtube.com/channel/SkwGlUSFoolH727C6OIwwA
**SqToEyltruYZs8tt-txmRg**	Université de Technologie de Belfort Montbéliard	https://www.youtube.com/channel/SqToEyltruYZs8tt-txmRg
**Sw6ZOMTstZu7oPWgXB7eqA**	Margot Velazquez	https://www.youtube.com/channel/Sw6ZOMTstZu7oPWgXB7eqA
**SwPcnzaMTuDcTgjRiJvZnw**	PCF - Parti communiste français	https://www.youtube.com/channel/SwPcnzaMTuDcTgjRiJvZnw
**Szx_YuvN_OpWRCjP83V4-Q**	lephoceen	https://www.youtube.com/channel/Szx_YuvN_OpWRCjP83V4-Q
**T67YOMntJxfRnO_9bXDpvw**	Le Média	https://www.youtube.com/channel/T67YOMntJxfRnO_9bXDpvw
**TCQjWrkWFtHhmA3YPOfijw**	Médecins du Monde	https://www.youtube.com/channel/TCQjWrkWFtHhmA3YPOfijw
**TFpJ5uRFY25i23Vd_--s8w**	Reservoir Apps	https://www.youtube.com/channel/TFpJ5uRFY25i23Vd_--s8w
**TItQw_aTIlt1i8ysgYMH3w**	Ministère de l'Europe et des Affaires étrangères	https://www.youtube.com/channel/TItQw_aTIlt1i8ysgYMH3w
**TOAKgwfqqCbOytF3RNGGVw**	Monsieur GRrr [FR]	https://www.youtube.com/channel/TOAKgwfqqCbOytF3RNGGVw
**TQQb3F6qDKXObGAE27HtVw**	Le Media Pour Tous	https://www.youtube.com/channel/TQQb3F6qDKXObGAE27HtVw
**TVh0Q7_mpSdU8b-V6kHxzg**	Patrick Le Hyaric	https://www.youtube.com/channel/TVh0Q7_mpSdU8b-V6kHxzg
**Ta1bPArjpvNMgSHAb_pflA**	Public Sénat	https://www.youtube.com/channel/Ta1bPArjpvNMgSHAb_pflA
**TafEJoRl5myC8A50pIlrng**	FabienOlicard	https://www.youtube.com/channel/TafEJoRl5myC8A50pIlrng
**TglyiuI7S_VZ4E3FinKs0w**	Pierre-Emmanuel Barré	https://www.youtube.com/channel/TglyiuI7S_VZ4E3FinKs0w
**TgMXsEqStBAcj69POnHvlQ**	François Jolivet	https://www.youtube.com/channel/TgMXsEqStBAcj69POnHvlQ
**TmqBtCRz42YAQOQO_--4hg**	Cécile UNTERMAIER	https://www.youtube.com/channel/TmqBtCRz42YAQOQO_--4hg
**TtMOVyHOKHp4i7lrnTfsiw**	Le Revenu TV	https://www.youtube.com/channel/TtMOVyHOKHp4i7lrnTfsiw
**U0FhLr6fr7U9GOn6OiQHpQ**	Officiel DEFAKATOR	https://www.youtube.com/channel/U0FhLr6fr7U9GOn6OiQHpQ
**U3z3px1_RCqYBwrs8LJVWg**	Marine Le Pen	https://www.youtube.com/channel/U3z3px1_RCqYBwrs8LJVWg
**UNhpUCY89yNhgpf05TpZ2w**	Elueslocales	https://www.youtube.com/channel/UNhpUCY89yNhgpf05TpZ2w
**UR03ZSZIEvCO6EWwv4jU2w**	Absol Vidéos	https://www.youtube.com/channel/UR03ZSZIEvCO6EWwv4jU2w
**UVKhb8NebymDohCBA_hT3w**	reinformation.tv	https://www.youtube.com/channel/UVKhb8NebymDohCBA_hT3w
**Ugq-zx8-w0qDTQoqyzo0-A**	on s'ennuie jeux et quizz	https://www.youtube.com/channel/Ugq-zx8-w0qDTQoqyzo0-A
**Uj4Kgn-R9kJZ96OWFB2P4g**	Passion Animale et végétale	https://www.youtube.com/channel/Uj4Kgn-R9kJZ96OWFB2P4g
**UsBMOIUl_ad6JUOC16DpmQ**	BFM Business	https://www.youtube.com/channel/UsBMOIUl_ad6JUOC16DpmQ
**UwtTJir4sZL06om7-KRl1A**	France 3 Centre-Val de Loire	https://www.youtube.com/channel/UwtTJir4sZL06om7-KRl1A
**UzYbvdW_HO7ykD39CAXB6Q**	C Pol	https://www.youtube.com/channel/UzYbvdW_HO7ykD39CAXB6Q
**Uzk9hBisz4aSMVTl37pWgw**	Lutte ouvrière	https://www.youtube.com/channel/Uzk9hBisz4aSMVTl37pWgw

**V-_U2U7SzI6obr4JXT4nxg**	ALLOGENIQUE	https://www.youtube.com/channel/V-_U2U7SzI6obr4JXT4nxg
**V4uoWB7m5Dl4VoUDn4GlXw**	Parents !	https://www.youtube.com/channel/V4uoWB7m5Dl4VoUDn4GlXw
**V8KMB7MpOAcbEfxp1bOuCA**	Galates	https://www.youtube.com/channel/V8KMB7MpOAcbEfxp1bOuCA
**V8hrENVRTTDQCHd5Vm_pVw**	Pacôme Rupin	https://www.youtube.com/channel/V8hrENVRTTDQCHd5Vm_pVw
**V9tcSCbEgwMUQdOGA1G5bQ**	Département Loire-Atlantique	https://www.youtube.com/channel/V9tcSCbEgwMUQdOGA1G5bQ
**VN2aPwAgQ-z5_56RdDrXSQ**	Jean-Manu	https://www.youtube.com/channel/VN2aPwAgQ-z5_56RdDrXSQ
**VNErcOrGeS5YT6ZWq0l1rA**	BLC TV	https://www.youtube.com/channel/VNErcOrGeS5YT6ZWq0l1rA
**VXiRA9Y7kTadGN_nlzt0Fg**	7 jours sur la planète	https://www.youtube.com/channel/VXiRA9Y7kTadGN_nlzt0Fg
**VYfSwcu7g_J344g4-AsMcw**	Eric Perroud 63	https://www.youtube.com/channel/VYfSwcu7g_J344g4-AsMcw
**VYiykzTUuEMg845nrkogSA**	Pas Végan	https://www.youtube.com/channel/VYiykzTUuEMg845nrkogSA
**VeMw72tepFl1Zt5fvf9QKQ**	Osons Causer	https://www.youtube.com/channel/VeMw72tepFl1Zt5fvf9QKQ
**Vfm2NnzYkNa0IHRFf3Relg**	Raphaël SCHELLENBERGER	https://www.youtube.com/channel/Vfm2NnzYkNa0IHRFf3Relg
**VjpxXJEDQb3Okjb-nV7oTg**	Freedom News Now	https://www.youtube.com/channel/VjpxXJEDQb3Okjb-nV7oTg
**Vq7AIRvJ0rDIV2ddHA7Emg**	Lozère-Nouvelle / 48info.fr	https://www.youtube.com/channel/Vq7AIRvJ0rDIV2ddHA7Emg
**VsMQmhMSj521lFSdXn5Pag**	Momo Rillon	https://www.youtube.com/channel/VsMQmhMSj521lFSdXn5Pag
**Vsw3Qrf2r7BiR-FesVMxtg**	TV Patriotes	https://www.youtube.com/channel/Vsw3Qrf2r7BiR-FesVMxtg
**Vz9cD4mvmpG4Nb_Rd2vxKw**	Philippe Folliot	https://www.youtube.com/channel/Vz9cD4mvmpG4Nb_Rd2vxKw
**W06vAptUNL6yGZFhkFrcAw**	Ben & Sav - Underground News	https://www.youtube.com/channel/W06vAptUNL6yGZFhkFrcAw
**W2QcKZiU8aUGg4yxCIditg**	euronews (en français)	https://www.youtube.com/channel/W2QcKZiU8aUGg4yxCIditg
**W3mewW-ZMdk3byz5F8gfHA**	France 3 Bretagne	https://www.youtube.com/channel/W3mewW-ZMdk3byz5F8gfHA
**WEVFCrnSVhCTcYBSDiWSNQ**	KILLUMINATY MASTA E.X OFFICIEL	https://www.youtube.com/channel/WEVFCrnSVhCTcYBSDiWSNQ
**WGorjF91vQIdb1gxEMXwQQ**	Licra	https://www.youtube.com/channel/WGorjF91vQIdb1gxEMXwQQ
**WWCQjtkZZB3Bb5zGKI41Nw**	Jacinte Sans h	https://www.youtube.com/channel/WWCQjtkZZB3Bb5zGKI41Nw
**Wc3I_Jt_YV3qtEvtvNOs-A**	Alexandre Lebreton	https://www.youtube.com/channel/Wc3I_Jt_YV3qtEvtvNOs-A
**WiO6cBQrCXLeuVdnO6F2Wg**	Solidarité et Progrès	https://www.youtube.com/channel/WiO6cBQrCXLeuVdnO6F2Wg
**WiQCbdC05zfzNg83F2kc3A**	France 3 Normandie	https://www.youtube.com/channel/WiQCbdC05zfzNg83F2kc3A
**WiR1j9tTALSQ-_0ax7HH1A**	Politique Assemblée	https://www.youtube.com/channel/WiR1j9tTALSQ-_0ax7HH1A
**Wk1e9QHIBU_582wRadfpoA**	Street Politics	https://www.youtube.com/channel/Wk1e9QHIBU_582wRadfpoA
**WtD8JN9hkxL5TJL_ktaNZA**	Grand Angle	https://www.youtube.com/channel/WtD8JN9hkxL5TJL_ktaNZA
**WtDTDMeHYVNlxuZt4NS5VA**	MCE TV	https://www.youtube.com/channel/WtDTDMeHYVNlxuZt4NS5VA
**Wyqks6hlvp0xrzgqp4T4EQ**	NEW CALEDONIA	https://www.youtube.com/channel/Wyqks6hlvp0xrzgqp4T4EQ
**X0p0BGwCfJoz7oB-TSJ57Q**	Jean-Pierre PETIT	https://www.youtube.com/channel/X0p0BGwCfJoz7oB-TSJ57Q
**X2dIyMEQMWy3AQBlq1CYXQ**	TéléCrayon	https://www.youtube.com/channel/X2dIyMEQMWy3AQBlq1CYXQ
**X74i0FzpGc5xothx7OTNAQ**	Vidgita Investigation	https://www.youtube.com/channel/X74i0FzpGc5xothx7OTNAQ
**X9lsdsTKfTi1eqoyL-RS-Q**	Mister Geopolitix	https://www.youtube.com/channel/X9lsdsTKfTi1eqoyL-RS-Q
**XHWT_QoQSsdGXbqF0hAMtg**	Arrêt sur Images	https://www.youtube.com/channel/XHWT_QoQSsdGXbqF0hAMtg
**XI6teCQRgNU3yAqmvTYZeQ**	ADBK TV	https://www.youtube.com/channel/XI6teCQRgNU3yAqmvTYZeQ
**XKJrYczY2_fJEZgFPGY0HQ**	CNEWS	https://www.youtube.com/channel/XKJrYczY2_fJEZgFPGY0HQ
**XUSLU65KYElvyrrbQA1KIw**	Faites entrer l'accusé	https://www.youtube.com/channel/XUSLU65KYElvyrrbQA1KIw
**Xbi34mIO6u_7qnUmK9FD9Q**	ArvalisTV	https://www.youtube.com/channel/Xbi34mIO6u_7qnUmK9FD9Q
**Xfp5IKQSutQvPlrDRKo9xg**	#Indecis	https://www.youtube.com/channel/Xfp5IKQSutQvPlrDRKo9xg
**XrGe_bC6X1RA5Zd8SNkugg**	Sicavonline	https://www.youtube.com/channel/XrGe_bC6X1RA5Zd8SNkugg
**XsmcfpNntkP5Mp2ZRKerDA**	Studyrama	https://www.youtube.com/channel/XsmcfpNntkP5Mp2ZRKerDA
**XtXCQhZXenA-3niEjcgnxg**	KEDISTAN	https://www.youtube.com/channel/XtXCQhZXenA-3niEjcgnxg
**XwDLMDV86ldKoFVc_g8P0g**	BFMTV	https://www.youtube.com/channel/XwDLMDV86ldKoFVc_g8P0g
**XwSQhtYH8a-HqDhNw3lNpQ**	Charles Demassieux	https://www.youtube.com/channel/XwSQhtYH8a-HqDhNw3lNpQ
**XzgD_oZ2jVOnEWNVDCnjjA**	RÉFRACTAIRE TV	https://www.youtube.com/channel/XzgD_oZ2jVOnEWNVDCnjjA
**Y9O4wYJYvgFoeWmfhtn-XA**	Farida Belghoul	https://www.youtube.com/channel/Y9O4wYJYvgFoeWmfhtn-XA
**YFI5PGuw_34XaA3SGDkQOQ**	Documentaire Société	https://www.youtube.com/channel/YFI5PGuw_34XaA3SGDkQOQ
**YH5iG-PNlvXtaypQYg0ziA**	Valérie Létard	https://www.youtube.com/channel/YH5iG-PNlvXtaypQYg0ziA
**YQNocWm-rzX6EULiasVh8g**	Génération Identitaire	https://www.youtube.com/channel/YQNocWm-rzX6EULiasVh8g
**YX4PcgqrQlx5togadd5JEA**	Croix-Rouge française	https://www.youtube.com/channel/YX4PcgqrQlx5togadd5JEA
**YdyGYM1aoLpAuCxsP86O1g**	Valek	https://www.youtube.com/channel/YdyGYM1aoLpAuCxsP86O1g
**YjbkXmibcm6JDjR7LeQk4w**	Kontre Kulture	https://www.youtube.com/channel/YjbkXmibcm6JDjR7LeQk4w

**YpRDnhk5H8h16jpS84uqsA**	Le Monde	https://www.youtube.com/channel/YpRDnhk5H8h16jpS84uqsA
**Yqz5THDdOQRtqrOD93rl-w**	VIGIER Philippe	https://www.youtube.com/channel/Yqz5THDdOQRtqrOD93rl-w
**YvQOj-t4u5-QKGB3AuoLjw**	Paris Match	https://www.youtube.com/channel/YvQOj-t4u5-QKGB3AuoLjw
**Z0_1glILhME6ZGZaucI4cA**	Vive L'Europe	https://www.youtube.com/channel/Z0_1glILhME6ZGZaucI4cA
**ZlCV2zfrY4hjZxjoohBOTA**	Audiovisuel Créativ'Arts	https://www.youtube.com/channel/ZlCV2zfrY4hjZxjoohBOTA
**ZPykzLX99tIw5FyVosNlvA**	Marguerite et les circonstances	https://www.youtube.com/channel/ZPykzLX99tIw5FyVosNlvA
**ZRkh4vCJjZ_6rehxm2KvMw**	François Cocq	https://www.youtube.com/channel/ZRkh4vCJjZ_6rehxm2KvMw
**Zb2wDd2b13luZN03HAKuHQ**	Aciiderixx	https://www.youtube.com/channel/Zb2wDd2b13luZN03HAKuHQ
**ZfVorL83NxkuNOks8XhN3w**	IMAZPRESS Réunion	https://www.youtube.com/channel/ZfVorL83NxkuNOks8XhN3w
**ZgJ_r_Ewlu1Ck-RzNCHuUQ**	Clémentine Autain	https://www.youtube.com/channel/ZgJ_r_Ewlu1Ck-RzNCHuUQ
**ZhT4s66VouNwiSJ_uMsx9A**	Député LABARONNE	https://www.youtube.com/channel/ZhT4s66VouNwiSJ_uMsx9A
**ZjVdOOQaHgeHb4KHYmj-BA**	Midol TV	https://www.youtube.com/channel/ZjVdOOQaHgeHb4KHYmj-BA
**ZksUgbZ-ba-JIwElepdMfw**	AIELLO	https://www.youtube.com/channel/ZksUgbZ-ba-JIwElepdMfw
**ZlVTkSkyBwdhrJc3rTCOCg**	Gabriel Serville	https://www.youtube.com/channel/ZlVTkSkyBwdhrJc3rTCOCg
**ZoVBT8G5EPGvjhmbexkQ7Q**	ATROPINE	https://www.youtube.com/channel/ZoVBT8G5EPGvjhmbexkQ7Q
**ZpMHojA3PtNe0ubshu84zQ**	MIASME TV	https://www.youtube.com/channel/ZpMHojA3PtNe0ubshu84zQ
**ZvgdmfEVhN_bkzQKpBsi2w**	TV Landes	https://www.youtube.com/channel/ZvgdmfEVhN_bkzQKpBsi2w
**_3XOdJHZjCO6dryFf2JaOw**	notre-planete.info	https://www.youtube.com/channel/_3XOdJHZjCO6dryFf2JaOw
**_4WpPcSfpxsSqA6hNpqJbg**	Tout Compte Fait	https://www.youtube.com/channel/_4WpPcSfpxsSqA6hNpqJbg
**_4hZxxgIzCvzdreCffmY5Q**	ernesto deupoinzero	https://www.youtube.com/channel/_4hZxxgIzCvzdreCffmY5Q
**_56vSO35nctESDan8agevg**	DeBunKer des Etoiles	https://www.youtube.com/channel/_56vSO35nctESDan8agevg
**_Bi0PTuGgOAxljcwqC8cWQ**	Hervé Gaïa	https://www.youtube.com/channel/_Bi0PTuGgOAxljcwqC8cWQ
**_GxHrDkwwADSaAjmXxqtXA**	France 3 Auvergne-Rhône-Alpes	https://www.youtube.com/channel/_GxHrDkwwADSaAjmXxqtXA
**_HM0XBWS4YsQOmBVWqHsa**	Le Compost	https://www.youtube.com/channel/_HM0XBWS4YsQOmBVWqHsa
**_NBvUCmnjCuYVKJYD2sSgw**	France 3 Corse ViaStella	https://www.youtube.com/channel/_NBvUCmnjCuYVKJYD2sSgw
**_XwdoOY4qxDiJk6Z4NJxcQ**	frexit upr	https://www.youtube.com/channel/_XwdoOY4qxDiJk6Z4NJxcQ
**_XzGXXB_dEhw6qai6rDJPQ**	DEGOUDEN TV	https://www.youtube.com/channel/_XzGXXB_dEhw6qai6rDJPQ
**_ZRsbnKHxw5vECIpswQvXA**	Mohamed Laqhila	https://www.youtube.com/channel/_ZRsbnKHxw5vECIpswQvXA
**_i-uMpN1IEyuoUGDq-dajQ**	Le Raptor	https://www.youtube.com/channel/_i-uMpN1IEyuoUGDq-dajQ
**_iuTGIGPWQBLGbd_TgwB-Q**	Toute l'Histoire	https://www.youtube.com/channel/_iuTGIGPWQBLGbd_TgwB-Q
**_jsZt6_tqdd2JaFXwQFNRg**	Mr. KITSUNÉ	https://www.youtube.com/channel/_jsZt6_tqdd2JaFXwQFNRg
**_ktPlUolPt0OLmbbKZs8fQ**	6Medias	https://www.youtube.com/channel/_ktPlUolPt0OLmbbKZs8fQ
**_majyzBb8HZV0wCmU4Z4Ag**	imineo	https://www.youtube.com/channel/_majyzBb8HZV0wCmU4Z4Ag
**_sZE1-jMW40NzMPI6rCrUQ**	Aufeminin	https://www.youtube.com/channel/_sZE1-jMW40NzMPI6rCrUQ
**_yjQfBPiWxHtvyZ-Qg_nOg**	Sputnik France	https://www.youtube.com/channel/_yjQfBPiWxHtvyZ-Qg_nOg
**a346_ck5QS_XMY6qbJyTwQ**	un tout petit Grain de Sable	https://www.youtube.com/channel/a346_ck5QS_XMY6qbJyTwQ
**a7Drb7rb5byDi4WaDeRvsg**	SUEZ France	https://www.youtube.com/channel/a7Drb7rb5byDi4WaDeRvsg
**a9qtGLeyCG38lhcSslV7LA**	Joël Nadon	https://www.youtube.com/channel/a9qtGLeyCG38lhcSslV7LA
**aA-wv2dobbgl8emqre7JAg**	EPOKA	https://www.youtube.com/channel/aA-wv2dobbgl8emqre7JAg
**aEvMWzk3LdCpMZnntkdYwQ**	AGFB I Muvrini	https://www.youtube.com/channel/aEvMWzk3LdCpMZnntkdYwQ
**aLQQlPF3FRgQq8yjfkbJ8A**	Marie-France Lorho	https://www.youtube.com/channel/aLQQlPF3FRgQq8yjfkbJ8A
**aNRgcgEtTlyOz53F3fRUlA**	Loïc Chaigneau - IHT	https://www.youtube.com/channel/aNRgcgEtTlyOz53F3fRUlA
**aOaiEf63XL_7cyaDLP1GiA**	philippe jandrok	https://www.youtube.com/channel/aOaiEf63XL_7cyaDLP1GiA
**aRhYJWBim3qMhWV4Z4r6Og**	MontceauNews	https://www.youtube.com/channel/aRhYJWBim3qMhWV4Z4r6Og
**aSzSkQ-2VDmeUIXggXrn5g**	Bérangère Abba	https://www.youtube.com/channel/aSzSkQ-2VDmeUIXggXrn5g
**aUWCM_KjHR_PmXWlfV9AYQ**	Christophe Alévèque	https://www.youtube.com/channel/aUWCM_KjHR_PmXWlfV9AYQ
**aViDbhP5cqvonHb0SgyGvA**	Cité de l'économie	https://www.youtube.com/channel/aViDbhP5cqvonHb0SgyGvA
**aW1GNPTmPPexs0Uhf-Tg9g**	hommes libres	https://www.youtube.com/channel/aW1GNPTmPPexs0Uhf-Tg9g
**alpEds7VFZkaPONOTW91Iw**	L'Essor de la Gendarmerie	https://www.youtube.com/channel/alpEds7VFZkaPONOTW91Iw
**anxaCp65Ph4E1sPcEiwRkw**	Hebdomadaire RIVAROL	https://www.youtube.com/channel/anxaCp65Ph4E1sPcEiwRkw
**aoumiqIQOtXxHu7e3T1ghg**	Florian Rouanet	https://www.youtube.com/channel/aoumiqIQOtXxHu7e3T1ghg
**aqUCTIgFDtMhBeKeeejrkA**	Institut des Libertés - IDL	https://www.youtube.com/channel/aqUCTIgFDtMhBeKeeejrkA
**ar0yo-PpeMk51m116826pQ**	Journal l'Humanité	https://www.youtube.com/channel/ar0yo-PpeMk51m116826pQ
**axyID_2weUMc95cJIWK9Nw**	Charlie Hebdo	https://www.youtube.com/channel/axyID_2weUMc95cJIWK9Nw

**azDBRKv_Bh6Gf0ebKbFRPQ**	France 3 Pays de la Loire	https://www.youtube.com/channel/azDBRKv_Bh6Gf0ebKbFRPQ
**b4UAZwZqS4a35FrBcLlMXA**	CANAL+	https://www.youtube.com/channel/b4UAZwZqS4a35FrBcLlMXA
**b5yz3kBRZ5_5_ZgJ4GTjGw**	Joachim Son-Forget	https://www.youtube.com/channel/b5yz3kBRZ5_5_ZgJ4GTjGw
**b9EmqspwO4fHZHvJdM38HA**	Telerama	https://www.youtube.com/channel/b9EmqspwO4fHZHvJdM38HA
**b9q8s0CFtGIBdotWoJIrTQ**	Francie	https://www.youtube.com/channel/b9q8s0CFtGIBdotWoJIrTQ
**bB4GDRH4moETWvCZEaXibA**	Frédéric Petit	https://www.youtube.com/channel/bB4GDRH4moETWvCZEaXibA
**bDGREHVNo1dCvfagYUAstw**	noisiveleT	https://www.youtube.com/channel/bDGREHVNo1dCvfagYUAstw
**bGOiY3MeW2qHuZl2irxw9Q**	ARTE Radio	https://www.youtube.com/channel/bGOiY3MeW2qHuZl2irxw9Q
**bIvrZlVoI2w4TcYa32Pvdw**	F D	https://www.youtube.com/channel/bIvrZlVoI2w4TcYa32Pvdw
**bJ7NE_CBVIoB8cO3iDbnJg**	Éditions Conversano	https://www.youtube.com/channel/bJ7NE_CBVIoB8cO3iDbnJg
**bMwGRm0Zqux90FR0ov_cIQ**	Zap Médias	https://www.youtube.com/channel/bMwGRm0Zqux90FR0ov_cIQ
**bZ0Csr9ow6Xjkdd542Omhw**	Le Télégramme	https://www.youtube.com/channel/bZ0Csr9ow6Xjkdd542Omhw
**beOjdCRgyn2rWT3-MArg5Q**	Presse Océan	https://www.youtube.com/channel/beOjdCRgyn2rWT3-MArg5Q
**bfM5Y60K8pVcHyWftz1g8w**	La Règle du jeu	https://www.youtube.com/channel/bfM5Y60K8pVcHyWftz1g8w
**bj2azYYE_MHl0NCeaoC_iQ**	Groupe MoDem et apparentés	https://www.youtube.com/channel/bj2azYYE_MHl0NCeaoC_iQ
**bjAWrghAsQnSG3xMVupnuw**	Délivrance	https://www.youtube.com/channel/bjAWrghAsQnSG3xMVupnuw
**bkkkl3z_3tGvuwu__pHeKg**	Citoyen NADOT, Député.	https://www.youtube.com/channel/bkkkl3z_3tGvuwu__pHeKg
**bn8O8WwMeoZsPRxgumfvAQ**	École normale supérieure - PSL	https://www.youtube.com/channel/bn8O8WwMeoZsPRxgumfvAQ
**bn9qyH43REDjEKfWb5Vr7g**	Marguerite Deprez-Audebert	https://www.youtube.com/channel/bn9qyH43REDjEKfWb5Vr7g
**bnOz_ZnAitFe3JGQ6Y2x8A**	Hubert JULIEN-LAFERRIÈRE - Député	https://www.youtube.com/channel/bnOz_ZnAitFe3JGQ6Y2x8A
**bq4WwLfbhRxvyVYOTYU25g**	UnDropDansLaMare	https://www.youtube.com/channel/bq4WwLfbhRxvyVYOTYU25g
**btViN1SKDXRNLnL0-gPkVA**	hubert mlt	https://www.youtube.com/channel/btViN1SKDXRNLnL0-gPkVA
**bw0leS3f-J-xPaNJzOmwew**	TV78 - La chaîne des Yvelines	https://www.youtube.com/channel/bw0leS3f-J-xPaNJzOmwew
**bywTYy6s06KnBWLeGJ_86A**	Antyss77	https://www.youtube.com/channel/bywTYy6s06KnBWLeGJ_86A
**bz10_l4y0g6tmCqb4j_0rg**	Le Globe 777 PROD CITIZEN NEWS	https://www.youtube.com/channel/bz10_l4y0g6tmCqb4j_0rg
**c4Qj54pmamfJ28yiccnQ5Q**	Nicole Dubré Chirat	https://www.youtube.com/channel/c4Qj54pmamfJ28yiccnQ5Q
**cEOhL8boLnXFcV31fcDDiw**	HelloHelloInfo	https://www.youtube.com/channel/cEOhL8boLnXFcV31fcDDiw
**cJx5SR--QoGMDvxBzNfrCw**	TVLaTribune	https://www.youtube.com/channel/cJx5SR--QoGMDvxBzNfrCw
**cP3HpPMKiQHsj7qDzu3q6g**	Marinette — Femmes et féminisme	https://www.youtube.com/channel/cP3HpPMKiQHsj7qDzu3q6g
**cQw_bxFfBLL0yk2JzBn06Q**	A Alexandre D Dall'ara	https://www.youtube.com/channel/cQw_bxFfBLL0yk2JzBn06Q
**cWitztM-ucG9TTDPjrasNQ**	L'actu Paris	https://www.youtube.com/channel/cWitztM-ucG9TTDPjrasNQ
**cdgG2S1KYa-aj1G2HzZqDA**	Georges J.	https://www.youtube.com/channel/cdgG2S1KYa-aj1G2HzZqDA
**ceAtrRuCUdsRSBU07GValA**	Vérino	https://www.youtube.com/channel/ceAtrRuCUdsRSBU07GValA
**cfLuU0NFT0ZRoQy4jk6GdA**	CGTN Français	https://www.youtube.com/channel/cfLuU0NFT0ZRoQy4jk6GdA
**ckz6n8QccTd6K_xdwKqa0A**	AFP	https://www.youtube.com/channel/ckz6n8QccTd6K_xdwKqa0A
**csw3rWMB_E73VEm_hoS36w**	Jean-Christophe Lagarde	https://www.youtube.com/channel/csw3rWMB_E73VEm_hoS36w
**cueC-4NWGuPFQKzQWn5heA**	Victor Ferry	https://www.youtube.com/channel/cueC-4NWGuPFQKzQWn5heA
**cziTK2NKeWtWQ6kB5tmQ8Q**	e-penser	https://www.youtube.com/channel/cziTK2NKeWtWQ6kB5tmQ8Q
**d2sODjpk-z-QDj8_j42oKg**	Les Déconnomistes	https://www.youtube.com/channel/d2sODjpk-z-QDj8_j42oKg
**d3UFlxPws9gxE6NKkGfWZA**	Jean Pierre PONT	https://www.youtube.com/channel/d3UFlxPws9gxE6NKkGfWZA
**d5DKToXYTKAQ6khzewww2g**	France Culture	https://www.youtube.com/channel/d5DKToXYTKAQ6khzewww2g
**dLWgmzlk7xX_pqpNy1ElfQ**	Nain deux trois	https://www.youtube.com/channel/dLWgmzlk7xX_pqpNy1ElfQ
**dfaFWwwA9Z4zhpcaqojdPw**	Whip.	https://www.youtube.com/channel/dfaFWwwA9Z4zhpcaqojdPw
**dijnb4OugCB6KUl2aN3qJQ**	Guyane la 1ère	https://www.youtube.com/channel/dijnb4OugCB6KUl2aN3qJQ
**dnaDhU-LDQrlEEmSIfq0-Q**	Mediapart	https://www.youtube.com/channel/dnaDhU-LDQrlEEmSIfq0-Q
**doZvj96JFBABnnsN2h1BDw**	BFM Lyon	https://www.youtube.com/channel/doZvj96JFBABnnsN2h1BDw
**dqoRecG2o9oI8CA-kx2Wpg**	Radio Athéna	https://www.youtube.com/channel/dqoRecG2o9oI8CA-kx2Wpg
**drpG0vavJxispg60gGPCAg**	Free Panther	https://www.youtube.com/channel/drpG0vavJxispg60gGPCAg
**duEo4UFNiIshAobFADVyWg**	Catherine Kamowski	https://www.youtube.com/channel/duEo4UFNiIshAobFADVyWg
**dwvgwMAV66OVckB07psJBg**	La chaine Terrienne	https://www.youtube.com/channel/dwvgwMAV66OVckB07psJBg
**dz511AnVKOYHckD6jXqGBw**	Christian Cotten	https://www.youtube.com/channel/dz511AnVKOYHckD6jXqGBw
**e1XerrT8b9HALB4s-ubPeg**	Opium57 mar0	https://www.youtube.com/channel/e1XerrT8b9HALB4s-ubPeg
**e27I-_81PEpsG_GlYPEBog**	Les députés communistes	https://www.youtube.com/channel/e27I-_81PEpsG_GlYPEBog
**e2_2k-_81Y4RNKBnlMjrLg**	Ministère de la Transition écologique et Solidaire	https://www.youtube.com/channel/e2_2k-_81Y4RNKBnlMjrLg

**e7IHC7FcvUacXnwIMj1sfg**	Le Fil d'Ariane #Garnault&Associés	https://www.youtube.com/channel/e7IHC7FcvUacXnwIMj1sfg
**e8Kqxbh4ZYNIToCisQC9Og**	La République de Seine-et-Marne	https://www.youtube.com/channel/e8Kqxbh4ZYNIToCisQC9Og
**eB-KfF4E4yWEluGQc7LK0g**	Banyulsinfo	https://www.youtube.com/channel/eB-KfF4E4yWEluGQc7LK0g
**eCO3SDaFKqAiEODHbhw9CQ**	La Commère 43	https://www.youtube.com/channel/eCO3SDaFKqAiEODHbhw9CQ
**eD8USxztgQDLPnUpMmTtkA**	Les Éveilleurs	https://www.youtube.com/channel/eD8USxztgQDLPnUpMmTtkA
**eHJdlr9EwdppvyhlK3ZEUg**	Nicolas Turquois	https://www.youtube.com/channel/eHJdlr9EwdppvyhlK3ZEUg
**eIfe01u41FBRyVFYW0_uHw**	Verino Classics	https://www.youtube.com/channel/eIfe01u41FBRyVFYW0_uHw
**eMZkZqZGmOXqZZVOPf8rPg**	Astrolabe	https://www.youtube.com/channel/eMZkZqZGmOXqZZVOPf8rPg
**eWMp4Frgyv275gSnWNYoZQ**	Rassemblement National	https://www.youtube.com/channel/eWMp4Frgyv275gSnWNYoZQ
**eXH7fpqGj-MCPPLpH_J3FQ**	Enquête d'Infos	https://www.youtube.com/channel/eXH7fpqGj-MCPPLpH_J3FQ
**eXsZz8C5sUZe1YgDxR6j9A**	Le Pixel Mort	https://www.youtube.com/channel/eXsZz8C5sUZe1YgDxR6j9A
**edj6kEkWXtL61opkb0Or2Q**	Marie Claire	https://www.youtube.com/channel/edj6kEkWXtL61opkb0Or2Q
**eeA4mvlV68ixG-BhIvUycQ**	Abu-Ayyub Cédric-Ali	https://www.youtube.com/channel/eeA4mvlV68ixG-BhIvUycQ
**egHY3YnuweGR-OYr82XG1A**	Actu-Environnement	https://www.youtube.com/channel/egHY3YnuweGR-OYr82XG1A
**egkqjfwwEIHVkLO6Dsy4ig**	Nouvelles Terre	https://www.youtube.com/channel/egkqjfwwEIHVkLO6Dsy4ig
**ehLcXRfxHHyMfNEBwFj3BQ**	Médecins Sans Frontières (MSF)	https://www.youtube.com/channel/ehLcXRfxHHyMfNEBwFj3BQ
**ejTWYquMXD_MFHRZiqo_Aw**	C à vous	https://www.youtube.com/channel/ejTWYquMXD_MFHRZiqo_Aw
**enLr8FR6fDvX5pztrRKILQ**	Bretagne Télé	https://www.youtube.com/channel/enLr8FR6fDvX5pztrRKILQ
**eoMxgbBbm9JdEez21bd1mw**	Discord Insoumis	https://www.youtube.com/channel/eoMxgbBbm9JdEez21bd1mw
**epKLEk_iOkSVaT0MdD5_9w**	Riposte Laïque 2	https://www.youtube.com/channel/epKLEk_iOkSVaT0MdD5_9w
**eqx-_VStNvoThSdTP8RZpQ**	DATMAN	https://www.youtube.com/channel/eqx-_VStNvoThSdTP8RZpQ
**ewhc0fvja891XkpIPGRMxQ**	LCI	https://www.youtube.com/channel/ewhc0fvja891XkpIPGRMxQ
**f-DN6QjyyEbhuMWCoXdXQQ**	Phonandroid	https://www.youtube.com/channel/f-DN6QjyyEbhuMWCoXdXQQ
**f4cED8rhDtMQO44iZ-3QpQ**	Cathy RACON-BOUZON	https://www.youtube.com/channel/f4cED8rhDtMQO44iZ-3QpQ
**fA5DnCDX3Ixy5QOAMGtBlA**	Nicolas Dupont-Aignan	https://www.youtube.com/channel/fA5DnCDX3Ixy5QOAMGtBlA
**fHAdU2VfudEZ1dEhv3JhFQ**	Lepetitjournalcom	https://www.youtube.com/channel/fHAdU2VfudEZ1dEhv3JhFQ
**fHn_8-ehdem86fEvlFg-Gw**	Le Parisien	https://www.youtube.com/channel/fHn_8-ehdem86fEvlFg-Gw
**fNDF4xXMl5L2r5GO_IdgqQ**	Fondation Jean-Jaurès	https://www.youtube.com/channel/fNDF4xXMl5L2r5GO_IdgqQ
**fQpT8ebZlk8PndKVNTiAvA**	Gouvernement	https://www.youtube.com/channel/fQpT8ebZlk8PndKVNTiAvA
**fQubFnNRbuWgOfCsQNlsAw**	koi de 9	https://www.youtube.com/channel/fQubFnNRbuWgOfCsQNlsAw
**fS5OjxwurfJ7bnB0LK8gCQ**	Sapiens sur un caillou	https://www.youtube.com/channel/fS5OjxwurfJ7bnB0LK8gCQ
**fSlxCj1Nn4lkPag7saW0yA**	OneWayOfficiel	https://www.youtube.com/channel/fSlxCj1Nn4lkPag7saW0yA
**f_l-z2U9SEo04BF0v035kw**	Potentia Multitudinis	https://www.youtube.com/channel/f_l-z2U9SEo04BF0v035kw
**fkV7TVyjL8CIJhBafkhMdA**	CCTV Français	https://www.youtube.com/channel/fkV7TVyjL8CIJhBafkhMdA
**fsTAnYne5szhoMc381A5wg**	L'Opinion	https://www.youtube.com/channel/fsTAnYne5szhoMc381A5wg
**ftH-SRv8YpskSaHPXuIKzA**	Université Paris Dauphine-PSL	https://www.youtube.com/channel/ftH-SRv8YpskSaHPXuIKzA
**ftjwa5w8mGK3DXbvdcYWcw**	Mediacord Productions Vidéo	https://www.youtube.com/channel/ftjwa5w8mGK3DXbvdcYWcw
**fxAKP2hCl9_WaQsYR2OjRg**	HYM MEDIA	https://www.youtube.com/channel/fxAKP2hCl9_WaQsYR2OjRg
**fzdsdUXCo4cp7wdnGqwPUg**	Gilbert Collard	https://www.youtube.com/channel/fzdsdUXCo4cp7wdnGqwPUg
**g54S6igBf1rXLjqQryGmaQ**	Atheos	https://www.youtube.com/channel/g54S6igBf1rXLjqQryGmaQ
**gA9V57lQlZFXkDfhVGTWSg**	Martinique la 1ère	https://www.youtube.com/channel/gA9V57lQlZFXkDfhVGTWSg
**gB15YG-QcfzlFnI1Xm-2xg**	ANTILLUMINATI KEMARL1FAM	https://www.youtube.com/channel/gB15YG-QcfzlFnI1Xm-2xg
**gC7E2YoTgUytC5IdFScqfQ**	UNICEF France	https://www.youtube.com/channel/gC7E2YoTgUytC5IdFScqfQ
**gDIK63PnVL6VqP2-eWZ0XA**	Média 25	https://www.youtube.com/channel/gDIK63PnVL6VqP2-eWZ0XA
**gGb7tN3tIH5_Kk05D1J_bA**	RMC	https://www.youtube.com/channel/gGb7tN3tIH5_Kk05D1J_bA
**gI52Zm1UcLxKD2Syepohjg**	Le Républicain Lorrain	https://www.youtube.com/channel/gI52Zm1UcLxKD2Syepohjg
**gOt-t1TagtT6MvsAg4ajiQ**	NURÉA TV - Au coeur de l'inexpliqué	https://www.youtube.com/channel/gOt-t1TagtT6MvsAg4ajiQ
**gPYsPyGhhQJQ9zOUQDWDMA**	i24NEWS Français	https://www.youtube.com/channel/gPYsPyGhhQJQ9zOUQDWDMA
**gVEQ0G3NvGCQDMIEvbRC8g**	Zehnor	https://www.youtube.com/channel/gVEQ0G3NvGCQDMIEvbRC8g
**gXIDZzs8ksN7k9pl3K-50Q**	GQ France	https://www.youtube.com/channel/gXIDZzs8ksN7k9pl3K-50Q
**gYqaZrPyWNEIEKOESjc8IA**	Action Française	https://www.youtube.com/channel/gYqaZrPyWNEIEKOESjc8IA
**gZqpf1zbwKAyD3IJ3OKDQQ**	Élu-e-s Génération-s Conseil de Paris	https://www.youtube.com/channel/gZqpf1zbwKAyD3IJ3OKDQQ
**goxc-VKU0cw9BcM9z_qokg**	Frandroid	https://www.youtube.com/channel/goxc-VKU0cw9BcM9z_qokg
**gtI_h-tB-MANQ722aNsboQ**	Catherine Fabre	https://www.youtube.com/channel/gtI_h-tB-MANQ722aNsboQ

**gv8ahGLkTVp4WyFK2-fymg**	Olivier SERVA	https://www.youtube.com/channel/gv8ahGLkTVp4WyFK2-fymg
**gvPhmp59vQ88z77v4KpcOQ**	Exister en Liberté	https://www.youtube.com/channel/gvPhmp59vQ88z77v4KpcOQ
**gwOaJC1E1EW1q0d9iRDSag**	Stella Dupont	https://www.youtube.com/channel/gwOaJC1E1EW1q0d9iRDSag
**gznYBXPpnoquWg91OKKlyQ**	Mehdi tous est rien	https://www.youtube.com/channel/gznYBXPpnoquWg91OKKlyQ
**h-U_EfevOIMmZmgXXtw9Gw**	BricoLePatriote	https://www.youtube.com/channel/h-U_EfevOIMmZmgXXtw9Gw
**h1D5qLzo-c_XWkFRsqFhtw**	Régis JUANICO	https://www.youtube.com/channel/h1D5qLzo-c_XWkFRsqFhtw
**h2YBKhYIy-_LtfCIn2Jycg**	Mr. Sam - Point d'interrogation	https://www.youtube.com/channel/h2YBKhYIy-_LtfCIn2Jycg
**h5JF-qt2ZXg8acejcJ15Xw**	Karambolage en français - ARTE	https://www.youtube.com/channel/h5JF-qt2ZXg8acejcJ15Xw
**h6aTwE8VczwBYkXmprvEAQ**	Le Stu-Dio	https://www.youtube.com/channel/h6aTwE8VczwBYkXmprvEAQ
**h8iNO5BcoyEPtKFNNotH5g**	Web FO	https://www.youtube.com/channel/h8iNO5BcoyEPtKFNNotH5g
**hDWHhc_vjr1xmQtbOS1-3g**	VOnews/vià95	https://www.youtube.com/channel/hDWHhc_vjr1xmQtbOS1-3g
**hF4zw3XZoJ6vQM1Lc7NDWQ**	Sarah El Haïry	https://www.youtube.com/channel/hF4zw3XZoJ6vQM1Lc7NDWQ
**hQeMsM8ux6kyq0WRncOerg**	Tsahal - Armée de Défense d'Israël	https://www.youtube.com/channel/hQeMsM8ux6kyq0WRncOerg
**hS22UTBbZTbb0L8hHR1P7A**	Benoit SIMIAN	https://www.youtube.com/channel/hS22UTBbZTbb0L8hHR1P7A
**hSBqxwkojyVQ7SZAPygOWA**	الاخوة_ لتعليم المبتدئين تربية وعلاج الطيور	https://www.youtube.com/channel/hSBqxwkojyVQ7SZAPygOWA
**hZR7a2ZeNngLp8GnYMWRtw**	CIWF France	https://www.youtube.com/channel/hZR7a2ZeNngLp8GnYMWRtw
**hZWs6PJY0hND384d2_RrhQ**	Les Echos	https://www.youtube.com/channel/hZWs6PJY0hND384d2_RrhQ
**hiWt-Y_fW2k0hVfgIFbe3Q**	instinCroma	https://www.youtube.com/channel/hiWt-Y_fW2k0hVfgIFbe3Q
**hjC3WOBMIm9QVVSEBtIkWA**	CLPRESS	https://www.youtube.com/channel/hjC3WOBMIm9QVVSEBtIkWA
**hq7IIJgKBr5cEID44Q6PIA**	Investig'Action	https://www.youtube.com/channel/hq7IIJgKBr5cEID44Q6PIA
**hqvR-HMW8ZMKjh02ufpVKg**	Jean-Michel Jacques	https://www.youtube.com/channel/hqvR-HMW8ZMKjh02ufpVKg
**huFDFG5lyLDEHrkXo7apEg**	Gallia - notre histoire de France	https://www.youtube.com/channel/huFDFG5lyLDEHrkXo7apEg
**i1h68Fys0apnhLaJ-ijQOw**	Ina Politique	https://www.youtube.com/channel/i1h68Fys0apnhLaJ-ijQOw
**i6trblthhr_E3oYLsFJBuw**	UNISSONS NOUS	https://www.youtube.com/channel/i6trblthhr_E3oYLsFJBuw
**i7b3afEzCptyJsU5i7ivNQ**	Elodie Hervé	https://www.youtube.com/channel/i7b3afEzCptyJsU5i7ivNQ
**i88JZlbs_C_BMfeAEAvnyA**	Philippe Berta	https://www.youtube.com/channel/i88JZlbs_C_BMfeAEAvnyA
**i9b1MGKmzqEDUxjWyy-p6A**	Préfecture Police	https://www.youtube.com/channel/i9b1MGKmzqEDUxjWyy-p6A
**iCzaDXmMcrW57Ky2_KcTHg**	La Grande Librairie	https://www.youtube.com/channel/iCzaDXmMcrW57Ky2_KcTHg
**iDGgCI8GxM9apVoqgKRDtQ**	L'Humanitaire dans tous ses états	https://www.youtube.com/channel/iDGgCI8GxM9apVoqgKRDtQ
**iJOjbmo2HJoOqiZZBDRT6w**	WWF France	https://www.youtube.com/channel/iJOjbmo2HJoOqiZZBDRT6w
**iK53gd_Da0ZlhUs0FP2CYQ**	Le Frexit	https://www.youtube.com/channel/iK53gd_Da0ZlhUs0FP2CYQ
**iKL3UdP0Zb58bOsKzSaS3g**	LA VOIX RACONTE	https://www.youtube.com/channel/iKL3UdP0Zb58bOsKzSaS3g
**iLEzPU-kdrfBZu_V3IPNCA**	Wallis-et-Futuna la 1ère	https://www.youtube.com/channel/iLEzPU-kdrfBZu_V3IPNCA
**iT4UyKh-cF8qBaBQP4p1ow**	Aurore Bergé	https://www.youtube.com/channel/iT4UyKh-cF8qBaBQP4p1ow
**iU8hILbqdubrjQ7qqXYxEg**	Majid Oukacha	https://www.youtube.com/channel/iU8hILbqdubrjQ7qqXYxEg
**iVFJiVl-GBPfmahaPZ4Hew**	Muriel Ressiguier	https://www.youtube.com/channel/iVFJiVl-GBPfmahaPZ4Hew
**iWYXqjnM7DPuI_WYsHkN4g**	Saber Solo Radio	https://www.youtube.com/channel/iWYXqjnM7DPuI_WYsHkN4g
**iWZjKH5VVJiYxJkUaVbZ9A**	ben voyons!	https://www.youtube.com/channel/iWZjKH5VVJiYxJkUaVbZ9A
**iZ005Ac5axBC9-p0Zz3GPQ**	TARANIS NEWS	https://www.youtube.com/channel/iZ005Ac5axBC9-p0Zz3GPQ
**ie3TTpnpck9Gy5dViMA_MQ**	La vérité est ailleurs	https://www.youtube.com/channel/ie3TTpnpck9Gy5dViMA_MQ
**ikITT772ffKI2zbZ5sX3Lg**	Draw my economy	https://www.youtube.com/channel/ikITT772ffKI2zbZ5sX3Lg
**ilxiEGEQHVZ25GU_cnyCzg**	Mathilde Panot	https://www.youtube.com/channel/ilxiEGEQHVZ25GU_cnyCzg
**inRP-BvNUmtu6inwENIWXw**	Ville de Nanterre	https://www.youtube.com/channel/inRP-BvNUmtu6inwENIWXw
**io9pX2dEW7mFncFsZLG6-g**	Guerre de Classe	https://www.youtube.com/channel/io9pX2dEW7mFncFsZLG6-g
**ixe3VYdKvKavOyopBxsKCw**	Place publique	https://www.youtube.com/channel/ixe3VYdKvKavOyopBxsKCw
**j0NPP7Q09tBDjQXVIMGvtw**	Cercle Aristote	https://www.youtube.com/channel/j0NPP7Q09tBDjQXVIMGvtw
**j1lGRXxXk2s-7yC1GQogwA**	Les Grosses Têtes	https://www.youtube.com/channel/j1lGRXxXk2s-7yC1GQogwA
**jA22pgPEWtIfTBiZunsW4g**	Grenoble Ecole de Management	https://www.youtube.com/channel/jA22pgPEWtIfTBiZunsW4g
**jCeyHQarZ0gtndcuz4dEkw**	Korben	https://www.youtube.com/channel/jCeyHQarZ0gtndcuz4dEkw
**jI5ayHwe-O3l42vwrJrMnw**	Le Partage	https://www.youtube.com/channel/jI5ayHwe-O3l42vwrJrMnw
**jRUOduxF-AlhU1y8P_pqLA**	La chaîne qui libère	https://www.youtube.com/channel/jRUOduxF-AlhU1y8P_pqLA
**jW0t60bMZ1fJcpD90vAoDQ**	Christian Person	https://www.youtube.com/channel/jW0t60bMZ1fJcpD90vAoDQ
**jWCDo8YqQb2tFFbQ1ZdacA**	Guy Fawkes	https://www.youtube.com/channel/jWCDo8YqQb2tFFbQ1ZdacA
**jY45gEsS77yul3XhQXD9iw**	REPORTAGE REPLAY	https://www.youtube.com/channel/jY45gEsS77yul3XhQXD9iw

**jaCN9r_oyIgyUwY7wgACkA**	Sciences Po	https://www.youtube.com/channel/jaCN9r_oyIgyUwY7wgACkA
**jaTrUFYwR0rbnrxJwmVHhA**	La Voix de Portici	https://www.youtube.com/channel/jaTrUFYwR0rbnrxJwmVHhA
**jcb1y2tnESxL19A2OX_APA**	Science & Vie TV	https://www.youtube.com/channel/jcb1y2tnESxL19A2OX_APA
**jgSf4vhWXgi3edYZiKrlig**	Dessine-moi l'éco	https://www.youtube.com/channel/jgSf4vhWXgi3edYZiKrlig
**jjqmADb2N34qTccsWgiAow**	Pieyre-Alexandre Anglade	https://www.youtube.com/channel/jjqmADb2N34qTccsWgiAow
**jjsC-0NuPc8YgqlmcrOMOw**	Alternatiba	https://www.youtube.com/channel/jjsC-0NuPc8YgqlmcrOMOw
**jlccQbBNBg8mJWdEri_DFw**	Le Courrier de l'Ouest	https://www.youtube.com/channel/jlccQbBNBg8mJWdEri_DFw
**juPrMBQjWlYwQUSxhuB1eQ**	Gamekult	https://www.youtube.com/channel/juPrMBQjWlYwQUSxhuB1eQ
**k-_PEY3iC6DIGJKuoEe9bw**	JEAN-LUC MÉLENCHON	https://www.youtube.com/channel/k-_PEY3iC6DIGJKuoEe9bw
**k0VjpVHbC0zu5KLXrHtpbw**	olivier-berruyer-les-crises	https://www.youtube.com/channel/k0VjpVHbC0zu5KLXrHtpbw
**k5a0Au3Lx883HBIaAMEzpw**	AmnestyFrance	https://www.youtube.com/channel/k5a0Au3Lx883HBIaAMEzpw
**kCIBdOQzWkSwkxvr5OaLGg**	VAR AZUR	https://www.youtube.com/channel/kCIBdOQzWkSwkxvr5OaLGg
**kIEMxZjqX_fiwSok0Uo9Pg**	Grand Lille TV	https://www.youtube.com/channel/kIEMxZjqX_fiwSok0Uo9Pg
**kJJzIl9NrWanvrdvLh_jbQ**	Après l'Effondrement	https://www.youtube.com/channel/kJJzIl9NrWanvrdvLh_jbQ
**kRr97IzIrXmynvq5YnveQQ**	CHEIKH DIENG OFFICIAL	https://www.youtube.com/channel/kRr97IzIrXmynvq5YnveQQ
**kV8sznD5D1P8ebLHdDl5ew**	Patrick LAROVERRE	https://www.youtube.com/channel/kV8sznD5D1P8ebLHdDl5ew
**kWDlmxaWWCdrCeLvY2VAsQ**	Le VRAI rassemblement	https://www.youtube.com/channel/kWDlmxaWWCdrCeLvY2VAsQ
**kZ3HK1sOLea8kIOqmIlBlA**	deodaTV	https://www.youtube.com/channel/kZ3HK1sOLea8kIOqmIlBlA
**k_gIrxQ67BHyIOSJOIZd4Q**	Les Pitches vous expliquent	https://www.youtube.com/channel/k_gIrxQ67BHyIOSJOIZd4Q
**kbAl6RbLWvT6ezX7_U1spQ**	GVS TV	https://www.youtube.com/channel/kbAl6RbLWvT6ezX7_U1spQ
**kbGwu2SKMZZOeodk3S26UA**	Marie Reno	https://www.youtube.com/channel/kbGwu2SKMZZOeodk3S26UA
**ke7Fgy2Jz9wKu_M5mC4K3A**	Maryline Richard	https://www.youtube.com/channel/ke7Fgy2Jz9wKu_M5mC4K3A
**keGNcjysxJr5dkCMQq0JEA**	Jean-Louis MASSON	https://www.youtube.com/channel/keGNcjysxJr5dkCMQq0JEA
**kiIklV0Nk46kCreyY47wzQ**	Anne Genetet	https://www.youtube.com/channel/kiIklV0Nk46kCreyY47wzQ
**klIMFT5cun08c1Wook2yQQ**	MIX ANTILLAISE	https://www.youtube.com/channel/klIMFT5cun08c1Wook2yQQ
**koBR7rxPUNE5rD-vk-PLuA**	Vincent Vauclin	https://www.youtube.com/channel/koBR7rxPUNE5rD-vk-PLuA
**kpOG3g2B7Ot5I6hIk84i4w**	Grégory ROOSE	https://www.youtube.com/channel/kpOG3g2B7Ot5I6hIk84i4w
**kywtKIAMrPEjddmOfdkZPA**	RAPOLITIK	https://www.youtube.com/channel/kywtKIAMrPEjddmOfdkZPA
**l7rD_6xuzuJOharNQzxItA**	Cercle des Volontaires	https://www.youtube.com/channel/l7rD_6xuzuJOharNQzxItA
**l8XXEKOg3r9du5Xb8zFcYQ**	Agora Des Savoirs	https://www.youtube.com/channel/l8XXEKOg3r9du5Xb8zFcYQ
**lA62pPS_ovEiLPtG5r6A1A**	Recrutement armée de Terre	https://www.youtube.com/channel/lA62pPS_ovEiLPtG5r6A1A
**lDk0iyj99stPxD9NQUaVSQ**	Espace des sciences	https://www.youtube.com/channel/lDk0iyj99stPxD9NQUaVSQ
**lEOrWPcIjrIsOksYNH2SZQ**	Léa Camilleri	https://www.youtube.com/channel/lEOrWPcIjrIsOksYNH2SZQ
**lF3ooWwdHouu74B60zvIVQ**	Journal Citoyen Haute-Marne	https://www.youtube.com/channel/lF3ooWwdHouu74B60zvIVQ
**lFzKs-2jaAEUem1Hp7hA4g**	paysdelaloire	https://www.youtube.com/channel/lFzKs-2jaAEUem1Hp7hA4g
**lJ5hcduPytNOiXKrVJLOOg**	Daniel Dupont	https://www.youtube.com/channel/lJ5hcduPytNOiXKrVJLOOg
**lJJQzmjn2cRb1KRXHRK0_w**	Les Jeunes Avec Macron	https://www.youtube.com/channel/lJJQzmjn2cRb1KRXHRK0_w
**lLFPD1pu0nVTR_Y8NCpXJQ**	Xerfi Canal	https://www.youtube.com/channel/lLFPD1pu0nVTR_Y8NCpXJQ
**lTsAerHYN_8ecbCtN8Wu7A**	Tout Simplement Ilian	https://www.youtube.com/channel/lTsAerHYN_8ecbCtN8Wu7A
**lVvloKPsVdo6vxqOSlqRuA**	SOS Racisme	https://www.youtube.com/channel/lVvloKPsVdo6vxqOSlqRuA
**laL6pKxFmsXFgd3LcrkvkQ**	Fréquence Occident	https://www.youtube.com/channel/laL6pKxFmsXFgd3LcrkvkQ
**laa_CwoQEmSo9Mb_M1f91g**	FLORIAN PHILIPPOT	https://www.youtube.com/channel/laa_CwoQEmSo9Mb_M1f91g
**li6Q1pd3DitknGEuMKnAVA**	Vogue Paris	https://www.youtube.com/channel/li6Q1pd3DitknGEuMKnAVA
**lpfQyL4qNSCAIr8NKoPRrA**	lesmardis	https://www.youtube.com/channel/lpfQyL4qNSCAIr8NKoPRrA
**lrZcn9PZV7hvNd0O_gsePA**	#DémarreTaStory avec l'apprentissage	https://www.youtube.com/channel/lrZcn9PZV7hvNd0O_gsePA
**lypba-Gixiu1nUyKbTE9qA**	MesFinances	https://www.youtube.com/channel/lypba-Gixiu1nUyKbTE9qA
**lz8i0_jW2wl3bBVE7vZuGg**	Savoie Le Département	https://www.youtube.com/channel/lz8i0_jW2wl3bBVE7vZuGg
**m-VXxCPg1zXQy8wjT0XKtA**	Marie-Ange MAGNE	https://www.youtube.com/channel/m-VXxCPg1zXQy8wjT0XKtA
**m4fSpMpPIZ9z6KHoezLUAg**	RFI	https://www.youtube.com/channel/m4fSpMpPIZ9z6KHoezLUAg
**m5wThREh298TkK8hCT9HuA**	Data Gueule	https://www.youtube.com/channel/m5wThREh298TkK8hCT9HuA
**m8-Ft8PdYGiSrB9cY2inDg**	Citoyen prévoyant	https://www.youtube.com/channel/m8-Ft8PdYGiSrB9cY2inDg
**mFF3pNeEzppnrZEf_rkmRg**	Télénantes	https://www.youtube.com/channel/mFF3pNeEzppnrZEf_rkmRg
**mHfCD8c8CmjlyWa8d4GObg**	Clique TV	https://www.youtube.com/channel/mHfCD8c8CmjlyWa8d4GObg
**mIakQqKbZn2gtaAqzGeRMw**	L'écho du peuple	https://www.youtube.com/channel/mIakQqKbZn2gtaAqzGeRMw

**mJvWpK-SYfXgf40MZNstUw**	l'Actu	https://www.youtube.com/channel/mJvWpK-SYfXgf40MZNstUw
**mLV2cNsYBQa6OvcyFQ3feg**	Jeunes IHEDN	https://www.youtube.com/channel/mLV2cNsYBQa6OvcyFQ3feg
**mPSwsooZq8an7xOLQQhAdw**	Rémi GAILLARD	https://www.youtube.com/channel/mPSwsooZq8an7xOLQQhAdw
**mWZqg1gUyouMUQuVmNXixQ**	Game & Intervention	https://www.youtube.com/channel/mWZqg1gUyouMUQuVmNXixQ
**mZ7YkJ_igDgSQck8fVnSdg**	HUB Institute	https://www.youtube.com/channel/mZ7YkJ_igDgSQck8fVnSdg
**mZk9OSmQ_5aw9iYh6xSzoA**	Université Paris 1 Panthéon-Sorbonne	https://www.youtube.com/channel/mZk9OSmQ_5aw9iYh6xSzoA
**mbhrKMU_X7Gi47bZEEajEQ**	StreetPress	https://www.youtube.com/channel/mbhrKMU_X7Gi47bZEEajEQ
**mcS4XR7iv1R4tB6EFmWgzA**	Hebdomadaire Réforme	https://www.youtube.com/channel/mcS4XR7iv1R4tB6EFmWgzA
**medNNXEe7V-nW6aoChUGiQ**	Valérie Oppelt	https://www.youtube.com/channel/medNNXEe7V-nW6aoChUGiQ
**minx_9bdEOPDmmLzUhpoSQ**	Corse-Matin Presse	https://www.youtube.com/channel/minx_9bdEOPDmmLzUhpoSQ
**mmABR5AlyLaQSINGa0Pz5Q**	Ciel Voilé	https://www.youtube.com/channel/mmABR5AlyLaQSINGa0Pz5Q
**mpeyZZqKDfUrtgCVVzfEjw**	Louis	https://www.youtube.com/channel/mpeyZZqKDfUrtgCVVzfEjw
**mvsy9pQaM6P1jdFZGLAmNg**	La Nouvelle République - NRCO	https://www.youtube.com/channel/mvsy9pQaM6P1jdFZGLAmNg
**mwxvryb7ZOJl_sl8R_L_gg**	Galixiss	https://www.youtube.com/channel/mwxvryb7ZOJl_sl8R_L_gg
**mzE9RqR_6_F6ib_yirMn2Q**	UFOTINIK	https://www.youtube.com/channel/mzE9RqR_6_F6ib_yirMn2Q
**n2qB6okVotIM0kW7tu3m4g**	Tariq Ramadan Official	https://www.youtube.com/channel/n2qB6okVotIM0kW7tu3m4g
**n8Kz9SiqdcAlrinWkgmtmQ**	HETZEL Patrick	https://www.youtube.com/channel/n8Kz9SiqdcAlrinWkgmtmQ
**n8zNIfYAQNdrFRrr8oibKw**	VICE	https://www.youtube.com/channel/n8zNIfYAQNdrFRrr8oibKw
**nDe3g44LrSqYZ4JN4TQLug**	Isadora Duncan	https://www.youtube.com/channel/nDe3g44LrSqYZ4JN4TQLug
**nIGrvSrQF7uCovnIm8LCSQ**	Demos Kratos	https://www.youtube.com/channel/nIGrvSrQF7uCovnIm8LCSQ
**nIWNujSpiiuH29-TBCz9aw**	Valeurs actuelles	https://www.youtube.com/channel/nIWNujSpiiuH29-TBCz9aw
**nJ8fyizHQi6A_5_5Im7cVA**	Boursorama	https://www.youtube.com/channel/nJ8fyizHQi6A_5_5Im7cVA
**nOQUorDkLzzMs9nsNG6DZg**	Gérard Cherpion	https://www.youtube.com/channel/nOQUorDkLzzMs9nsNG6DZg
**nQwKJLT0q8VypJURCnoTyA**	Des illusions	https://www.youtube.com/channel/nQwKJLT0q8VypJURCnoTyA
**nYYI0quQ5ZQ2ESnxcUk9gw**	Télé7 jours	https://www.youtube.com/channel/nYYI0quQ5ZQ2ESnxcUk9gw
**n_smTYmROafIdN_Ykomy8w**	France 3 Toutes Régions	https://www.youtube.com/channel/n_smTYmROafIdN_Ykomy8w
**ndDMMpugVtAbDKRbRI4glA**	Wake Up Rappel Dans l'Dine	https://www.youtube.com/channel/ndDMMpugVtAbDKRbRI4glA
**nj0nFFXDXoY0SuNps8PrVA**	Mairie de Toulouse	https://www.youtube.com/channel/nj0nFFXDXoY0SuNps8PrVA
**nmfwrOPcwNTiizizVb4VyA**	Christo Infos	https://www.youtube.com/channel/nmfwrOPcwNTiizizVb4VyA
**nnFyyCV7MchYtswi87AR8w**	Ministère Territoires et Collectivités	https://www.youtube.com/channel/nnFyyCV7MchYtswi87AR8w
**nrJMmhoVtOIbfq7hbbUq6w**	yanndriks	https://www.youtube.com/channel/nrJMmhoVtOIbfq7hbbUq6w
**nvygkFyEyIXZlG7tjCfMpw**	Michel Onfray TV	https://www.youtube.com/channel/nvygkFyEyIXZlG7tjCfMpw
**o3AhnfsCnyDF9Z73MA0q-w**	Gilles Lartigot	https://www.youtube.com/channel/o3AhnfsCnyDF9Z73MA0q-w
**oORK5hvfsEpTQlQEzsvINw**	jim le reveilleur	https://www.youtube.com/channel/oORK5hvfsEpTQlQEzsvINw
**oPZ-VcTFfkb-VlhsfzYp0Q**	On n'est plus des pigeons !	https://www.youtube.com/channel/oPZ-VcTFfkb-VlhsfzYp0Q
**oPik8tPlQKUtZuqQoM63cg**	Paul Molac	https://www.youtube.com/channel/oPik8tPlQKUtZuqQoM63cg
**oQa3Zf8ntJ1V-L4d6saVhw**	Stratpol	https://www.youtube.com/channel/oQa3Zf8ntJ1V-L4d6saVhw
**oRnHlbVByoYV6st5kPxOIQ**	Zap Télé Officiel	https://www.youtube.com/channel/oRnHlbVByoYV6st5kPxOIQ
**oSLEsFeBbVcEL1TZPartwQ**	Sylvain Waserman	https://www.youtube.com/channel/oSLEsFeBbVcEL1TZPartwQ
**oUFWjOMVsjN36m13jhrdPA**	Stef2892	https://www.youtube.com/channel/oUFWjOMVsjN36m13jhrdPA
**oV6J24qg5SZKVHAQzqhIUg**	IRD	https://www.youtube.com/channel/oV6J24qg5SZKVHAQzqhIUg
**odS0mi_n6wiArx4jJ32-kA**	Stéphane Blet	https://www.youtube.com/channel/odS0mi_n6wiArx4jJ32-kA
**oedwLMiVgvSknCpnJw7o6g**	KAMIIKAZ EN CLAQUETTE	https://www.youtube.com/channel/oedwLMiVgvSknCpnJw7o6g
**oid2lAJh8xEFWRaJ86S44w**	Michel VIALAY	https://www.youtube.com/channel/oid2lAJh8xEFWRaJ86S44w
**okvaOdBBaFspH2VHi0CDLA**	Herve duranto	https://www.youtube.com/channel/okvaOdBBaFspH2VHi0CDLA
**oouihyurnQjoDnQLTAcDZw**	Olivia Gregoire	https://www.youtube.com/channel/oouihyurnQjoDnQLTAcDZw
**owMG5mJqgrqitF4EnZPutA**	Ac Floraison	https://www.youtube.com/channel/owMG5mJqgrqitF4EnZPutA
**p2kK3DyhpgFOdILF3BBC9A**	L'Express	https://www.youtube.com/channel/p2kK3DyhpgFOdILF3BBC9A
**p6nnX0Zz1DRHtdLJlyfAaw**	Objectif Eco TV	https://www.youtube.com/channel/p6nnX0Zz1DRHtdLJlyfAaw
**p8CElQTorSmhH_jVgSSv5g**	Ecran Large	https://www.youtube.com/channel/p8CElQTorSmhH_jVgSSv5g
**pHdWsRO1_sotxnMtLL26VQ**	Keyholes & Snapshots	https://www.youtube.com/channel/pHdWsRO1_sotxnMtLL26VQ
**pQ4STl8j62KeiApns4FndA**	Députée Obono	https://www.youtube.com/channel/pQ4STl8j62KeiApns4FndA
**pTMWY8NRQby8zXUKhHznfg**	CEA Recherche	https://www.youtube.com/channel/pTMWY8NRQby8zXUKhHznfg
**pUcqKCtQybPvk0GmOv9u7Q**	Fannette Charvier	https://www.youtube.com/channel/pUcqKCtQybPvk0GmOv9u7Q

**pYE4jfZcrTr-AVBohx1_-g**	Adrien Quatennens	https://www.youtube.com/channel/pYE4jfZcrTr-AVBohx1_-g
**pZeCOYtTT-O1t9msb9zrDg**	Bruno Gollnisch	https://www.youtube.com/channel/pZeCOYtTT-O1t9msb9zrDg
**paOmN-F1fiS19Qh9vI8gAw**	1ère outre-mer	https://www.youtube.com/channel/paOmN-F1fiS19Qh9vI8gAw
**pbP0BfSEyu3_l-1u3bL3yg**	Mikaël Roparz	https://www.youtube.com/channel/pbP0BfSEyu3_l-1u3bL3yg
**pcd7UA71-eBeJnWftXx9HA**	Draw my news	https://www.youtube.com/channel/pcd7UA71-eBeJnWftXx9HA
**pfmGY18T_RqXfBgUB2Thsw**	Kevin Razy	https://www.youtube.com/channel/pfmGY18T_RqXfBgUB2Thsw
**pg4Z3tffxA4yMh4_0dFLbw**	Territoires!	https://www.youtube.com/channel/pg4Z3tffxA4yMh4_0dFLbw
**pi-4daExRSchtsJzO06rwA**	Quotidien	https://www.youtube.com/channel/pi-4daExRSchtsJzO06rwA
**piRUwO-qgZy8NyLJXi8W-A**	Jean-Marie Le Pen	https://www.youtube.com/channel/piRUwO-qgZy8NyLJXi8W-A
**pmXvJllrtbFKR846oKyJYg**	NygmaticK	https://www.youtube.com/channel/pmXvJllrtbFKR846oKyJYg
**pqPJXwNpJRt3xcD2n-PdsQ**	L'Aile à Stick	https://www.youtube.com/channel/pqPJXwNpJRt3xcD2n-PdsQ
**pv8Xt9IzKZjzfxBngP2bVQ**	Région Occitanie	https://www.youtube.com/channel/pv8Xt9IzKZjzfxBngP2bVQ
**pwWIg7cSrFja9MQgtdwBkw**	Brest Buzz	https://www.youtube.com/channel/pwWIg7cSrFja9MQgtdwBkw
**q-8pBMM3I40QlrhM9ExXJQ**	La Tronche en Biais	https://www.youtube.com/channel/q-8pBMM3I40QlrhM9ExXJQ
**q1PNGjQv-i2UvPR99XYWnQ**	Daniel CELLERIER	https://www.youtube.com/channel/q1PNGjQv-i2UvPR99XYWnQ
**q80IvL314jsE7PgYsTdw7Q**	Accropolis Replays	https://www.youtube.com/channel/q80IvL314jsE7PgYsTdw7Q
**q9xMn8vudIEJpJ_orJryRg**	Les Observateurs France 24	https://www.youtube.com/channel/q9xMn8vudIEJpJ_orJryRg
**qDb05AFZoC0_LH96na8qqQ**	Brefnews info	https://www.youtube.com/channel/qDb05AFZoC0_LH96na8qqQ
**qEVwTnDzlzKOGYNFemqnYA**	RT France	https://www.youtube.com/channel/qEVwTnDzlzKOGYNFemqnYA
**qEwL9lUdH-ccrN4Rgd0sEw**	Acrimed Vidéos	https://www.youtube.com/channel/qEwL9lUdH-ccrN4Rgd0sEw
**qFihsCXwjNs9hyHmEAACCw**	Jean-Charles Larsonneur	https://www.youtube.com/channel/qFihsCXwjNs9hyHmEAACCw
**qM2KCivU8WXj1_FWqoxIDg**	Réseau International	https://www.youtube.com/channel/qM2KCivU8WXj1_FWqoxIDg
**qOJZaD9IlpgMwSi-qANseQ**	Michelle Tirone	https://www.youtube.com/channel/qOJZaD9IlpgMwSi-qANseQ
**qRSWOqkW_oelrdUN7KQ-aQ**	Les Numériques	https://www.youtube.com/channel/qRSWOqkW_oelrdUN7KQ-aQ
**qWrfB18eAwY0UoQrCHLd5w**	Georgia Pouliquen	https://www.youtube.com/channel/qWrfB18eAwY0UoQrCHLd5w
**qaZpAnjhuNJMjk4f68QJSw**	Putsch Media	https://www.youtube.com/channel/qaZpAnjhuNJMjk4f68QJSw
**qapAnA17Im3bnYOpYXOcjA**	francetv slash / causes	https://www.youtube.com/channel/qapAnA17Im3bnYOpYXOcjA
**qfhEBf-OXhwW1DCoeffUWg**	Forum France	https://www.youtube.com/channel/qfhEBf-OXhwW1DCoeffUWg
**qm_zi4CmgAs_CySfciiEsQ**	V garou	https://www.youtube.com/channel/qm_zi4CmgAs_CySfciiEsQ
**qt99sKYNTxqlHtzV9weUYA**	VU FranceTV	https://www.youtube.com/channel/qt99sKYNTxqlHtzV9weUYA
**qv_wXmLSFtTDA39HQaLssQ**	Accropolis	https://www.youtube.com/channel/qv_wXmLSFtTDA39HQaLssQ
**qwac9xog45LRWMvufXs3hA**	Sputnik France Play	https://www.youtube.com/channel/qwac9xog45LRWMvufXs3hA
**qxXM5u3U1jwrI36fSB9VuA**	Gendarmerie Nationale	https://www.youtube.com/channel/qxXM5u3U1jwrI36fSB9VuA
**qySDfPcmKYq6oUeC03y57A**	vanessa beeley	https://www.youtube.com/channel/qySDfPcmKYq6oUeC03y57A
**r3SP1aBjElNUv5jqnuU2Vg**	David Azencot	https://www.youtube.com/channel/r3SP1aBjElNUv5jqnuU2Vg
**rJ-lE1Ah7IqLDLNnKFQa-g**	H&M	https://www.youtube.com/channel/rJ-lE1Ah7IqLDLNnKFQa-g
**rJSVVbj4EmhwzlBtEFzxwg**	Rue89 Strasbourg	https://www.youtube.com/channel/rJSVVbj4EmhwzlBtEFzxwg
**rL-mCKoPPh9NgR2rAM3kPw**	Economistes Atterrés	https://www.youtube.com/channel/rL-mCKoPPh9NgR2rAM3kPw
**rP3-mdG7xukDyDHHuFTzQg**	Marine Nationale	https://www.youtube.com/channel/rP3-mdG7xukDyDHHuFTzQg
**rPtG2PyP3pSxggR2vG6Gxw**	Chimical Spray	https://www.youtube.com/channel/rPtG2PyP3pSxggR2vG6Gxw
**rRKlEDktKeraMTgAN_DIzw**	Le Monde De Jamy	https://www.youtube.com/channel/rRKlEDktKeraMTgAN_DIzw
**rUplUpkwMXBFMQt3ohF07A**	Monuk	https://www.youtube.com/channel/rUplUpkwMXBFMQt3ohF07A
**rV-DjU6UivdNHOaazLttuQ**	La Renaissance du Bessin	https://www.youtube.com/channel/rV-DjU6UivdNHOaazLttuQ
**rYu3ROnolwmVTkLG-VBwFA**	Collège Européen	https://www.youtube.com/channel/rYu3ROnolwmVTkLG-VBwFA
**r_3nQ4eRCwm_XUDpf62MAg**	Partager C'est Sympa	https://www.youtube.com/channel/r_3nQ4eRCwm_XUDpf62MAg
**r_Oz6MFGScoquaZDKdjD-A**	le stUDIo	https://www.youtube.com/channel/r_Oz6MFGScoquaZDKdjD-A
**raJnhleOyGrEUMJSwbKJ8g**	Dino cinieri	https://www.youtube.com/channel/raJnhleOyGrEUMJSwbKJ8g
**rbbgYjbrXVYRM_iRGEDqFw**	Nota Bonus	https://www.youtube.com/channel/rbbgYjbrXVYRM_iRGEDqFw
**rmTJj_szl69W5ghhXg-SCA**	CitoiCitoyen	https://www.youtube.com/channel/rmTJj_szl69W5ghhXg-SCA
**rpsdF7UreiF-2r5Jp0YpRg**	Ouest-France	https://www.youtube.com/channel/rpsdF7UreiF-2r5Jp0YpRg
**rtnRJI-h6Rs9_6HOz3niBQ**	LES DÉCAFÉINÉS - Le Duo	https://www.youtube.com/channel/rtnRJI-h6Rs9_6HOz3niBQ
**ru8dCR1C-I9W2wIZw7ZIIA**	Ça Zap Tweet !	https://www.youtube.com/channel/ru8dCR1C-I9W2wIZw7ZIIA
**s-e7ayRI75k-i617JkCrpA**	Ina Culture	https://www.youtube.com/channel/s-e7ayRI75k-i617JkCrpA
**s46RDmgQFsMYg0H_eEBcfw**	Chambre de Commerce et d'Industrie de l'Ardèche	https://www.youtube.com/channel/s46RDmgQFsMYg0H_eEBcfw

**s4GI4gnPXdN7i3wSUm-Lpw**	L'esprit Viking - Oleg	https://www.youtube.com/channel/s4GI4gnPXdN7i3wSUm-Lpw
**sAoO_SdTB55bnb0aXHnxNQ**	lepopulaire.fr	https://www.youtube.com/channel/sAoO_SdTB55bnb0aXHnxNQ
**sGs7XjHyYuaBUoNnTF6k7Q**	LDC News Agency	https://www.youtube.com/channel/sGs7XjHyYuaBUoNnTF6k7Q
**sHW5CMU8QvPMPEXX7AtdzQ**	sambo 82	https://www.youtube.com/channel/sHW5CMU8QvPMPEXX7AtdzQ
**sHd-E4Q3hCgp_fvOf-tBiw**	Ina Humour	https://www.youtube.com/channel/sHd-E4Q3hCgp_fvOf-tBiw
**sLvt4E3ZXL8bakDz3jU77Q**	Aminégociateur TV aminegociateur	https://www.youtube.com/channel/sLvt4E3ZXL8bakDz3jU77Q
**sNC6taLBV0BqTPnDtkMNmA**	STEPHANE PARIS	https://www.youtube.com/channel/sNC6taLBV0BqTPnDtkMNmA
**sNdfYQciys2IAdAQpjFtUA**	Missor	https://www.youtube.com/channel/sNdfYQciys2IAdAQpjFtUA
**sdVv1p9XQ_8j-A1K3AgZWA**	Sophie Panonacle	https://www.youtube.com/channel/sdVv1p9XQ_8j-A1K3AgZWA
**sj_wRVDgEUXpiDbFXoC0TQ**	Libre Opinion	https://www.youtube.com/channel/sj_wRVDgEUXpiDbFXoC0TQ
**skzJ1Wu9FmcAbnEHJvWOgg**	9,999,999 Views	https://www.youtube.com/channel/skzJ1Wu9FmcAbnEHJvWOgg
**sua55kDzTYX_jztd7PckWw**	Aldo Sterone	https://www.youtube.com/channel/sua55kDzTYX_jztd7PckWw
**t2GVK8Qy7UGTkgssSTWHcQ**	MeilleurPlacement	https://www.youtube.com/channel/t2GVK8Qy7UGTkgssSTWHcQ
**t8ctlakIflnSG0ebFps7cw**	Histony	https://www.youtube.com/channel/t8ctlakIflnSG0ebFps7cw
**t9IC3dmZh-SlzGalndu5fQ**	Jugames V	https://www.youtube.com/channel/t9IC3dmZh-SlzGalndu5fQ
**tBZnQ0hzKfgaOrwSMsqavw**	Julien LAMOURETTE	https://www.youtube.com/channel/tBZnQ0hzKfgaOrwSMsqavw
**tCH6LGVdxn4QS9ty8YU9iQ**	News Infos Free	https://www.youtube.com/channel/tCH6LGVdxn4QS9ty8YU9iQ
**tJk8WNzhAu37r-QBmY7pRA**	La Croix	https://www.youtube.com/channel/tJk8WNzhAu37r-QBmY7pRA
**tMNeiW7rOdKOXhCexxQAyA**	Madame Figaro	https://www.youtube.com/channel/tMNeiW7rOdKOXhCexxQAyA
**tNA_FtemZa-EJeh8CAyJFQ**	Guillemant Philippe	https://www.youtube.com/channel/tNA_FtemZa-EJeh8CAyJFQ
**tNyuN_HFlNYO0-gqXwhLTg**	Fréquence Médicale	https://www.youtube.com/channel/tNyuN_HFlNYO0-gqXwhLTg
**tRcramlWbeFGR8RwpMRVpg**	Réseau Sortir du nucléaire	https://www.youtube.com/channel/tRcramlWbeFGR8RwpMRVpg
**tUBSLBp2wBYN4ckR-CiJWw**	video expresso	https://www.youtube.com/channel/tUBSLBp2wBYN4ckR-CiJWw
**tWXFE64qiX8k6CUER8zUgA**	Inform'Action	https://www.youtube.com/channel/tWXFE64qiX8k6CUER8zUgA
**tg3B1mxTUXSO30nT9azYGw**	GiletsJaunes Constituants	https://www.youtube.com/channel/tg3B1mxTUXSO30nT9azYGw
**tpBMYI8_OJvtydY3-zU-4A**	Anne-France Brunet Députée	https://www.youtube.com/channel/tpBMYI8_OJvtydY3-zU-4A
**tpRdVgkgUChGBIBGDUWgWA**	ET BIM	https://www.youtube.com/channel/tpRdVgkgUChGBIBGDUWgWA
**tqICqGbPSbTN09K1_7VZ3Q**	DirtyBiology	https://www.youtube.com/channel/tqICqGbPSbTN09K1_7VZ3Q
**ts9IsgQcfPyK-VCxZ8qxMA**	ESCLAVE HORROR STORY	https://www.youtube.com/channel/ts9IsgQcfPyK-VCxZ8qxMA
**ttrZYux2uArhx0SJ79vSfg**	Zonebourse	https://www.youtube.com/channel/ttrZYux2uArhx0SJ79vSfg
**tu9RkUwfQehjGTd_LhYJbg**	Batdaf 1	https://www.youtube.com/channel/tu9RkUwfQehjGTd_LhYJbg
**tyONQLs6htK-wWiCAk4wVw**	le Stagirite	https://www.youtube.com/channel/tyONQLs6htK-wWiCAk4wVw
**tyYjpiVPf6jJ020Fvn4ALA**	Bruno Le Salé	https://www.youtube.com/channel/tyYjpiVPf6jJ020Fvn4ALA
**tyZ1cGRbRnTGKok6RU9CwA**	L'invité	https://www.youtube.com/channel/tyZ1cGRbRnTGKok6RU9CwA
**u4nws5yvkvbcFUjDEh3cgw**	Conseil économique social et environnemental	https://www.youtube.com/channel/u4nws5yvkvbcFUjDEh3cgw
**u6iDp8QMjBfREopvVEX7Tw**	Nicolas MJ - Stratégie	https://www.youtube.com/channel/u6iDp8QMjBfREopvVEX7Tw
**u98K_ZNf4C_PFbfv05o4Kw**	UFO Conscience	https://www.youtube.com/channel/u98K_ZNf4C_PFbfv05o4Kw
**uBj_tnQHh0RHXiJxPkwBTA**	POSITIVR	https://www.youtube.com/channel/uBj_tnQHh0RHXiJxPkwBTA
**uBzVunAVbcwzMfQaJVacuw**	LE ROI DES RATS	https://www.youtube.com/channel/uBzVunAVbcwzMfQaJVacuw
**uE72HIKANo2IvS6EIDJ7EQ**	La Foi	https://www.youtube.com/channel/uE72HIKANo2IvS6EIDJ7EQ
**uENvZBO2yApXWP71mIuk9w**	Charente Libre	https://www.youtube.com/channel/uENvZBO2yApXWP71mIuk9w
**uPPPjRV7WyRGnfjK7_rvpA**	Commandant AUBENAS	https://www.youtube.com/channel/uPPPjRV7WyRGnfjK7_rvpA
**uQHD9MgnRILjwdQaWZrBxA**	ENGIE	https://www.youtube.com/channel/uQHD9MgnRILjwdQaWZrBxA
**uT7gfhmGtqo9biEbgfVmIw**	Melis Ann	https://www.youtube.com/channel/uT7gfhmGtqo9biEbgfVmIw
**uVBBWBdvnOZx2XU-TgLjxA**	La Française	https://www.youtube.com/channel/uVBBWBdvnOZx2XU-TgLjxA
**uViTVc1yXoIbBodmzZajFw**	Ministère de la Justice	https://www.youtube.com/channel/uViTVc1yXoIbBodmzZajFw
**uVx9duCZYExp4Z4PNhzPXw**	jordanix	https://www.youtube.com/channel/uVx9duCZYExp4Z4PNhzPXw
**uXJ4qKWpik3B6HDb3-mrbw**	koi de neuf?	https://www.youtube.com/channel/uXJ4qKWpik3B6HDb3-mrbw
**uYN15i2J_gETGamUAjEIWA**	RADIO RCJ	https://www.youtube.com/channel/uYN15i2J_gETGamUAjEIWA
**uYN7dG7X7gD7pEKcRsBZEA**	ERTV Officiel	https://www.youtube.com/channel/uYN7dG7X7gD7pEKcRsBZEA
**uZaaQbKpl_6R10VSZ--nOg**	independenzawebtv	https://www.youtube.com/channel/uZaaQbKpl_6R10VSZ--nOg
**uiEON01g2bUVEwIPCLzwRw**	Greg empêche moi	https://www.youtube.com/channel/uiEON01g2bUVEwIPCLzwRw
**uih8x8eQDAfA2J3H_BrYAQ**	Nouvelles Générales	https://www.youtube.com/channel/uih8x8eQDAfA2J3H_BrYAQ
**ukbnzZSfOVmszwwHGK7tqw**	LaBajon	https://www.youtube.com/channel/ukbnzZSfOVmszwwHGK7tqw

**ulwmNtmncNpqTEqKd0kZaQ**	Born2Heal	https://www.youtube.com/channel/ulwmNtmncNpqTEqKd0kZaQ
**uxVPSO0_EiL01_tneRCEcA**	David, de la chaîne Ganesh2	https://www.youtube.com/channel/uxVPSO0_EiL01_tneRCEcA
**v0AgTk5bBtqBTjsRcQtMcA**	Cédric Villani	https://www.youtube.com/channel/v0AgTk5bBtqBTjsRcQtMcA
**v0KbaxD9MdQ-V_sHG6eswg**	Retransmission	https://www.youtube.com/channel/v0KbaxD9MdQ-V_sHG6eswg
**v7I-SNC4Con0BfdLEWHUPA**	Riposte Laïque	https://www.youtube.com/channel/v7I-SNC4Con0BfdLEWHUPA
**vBbqBXCnsv8uU0UIfS1DBQ**	SACR TV	https://www.youtube.com/channel/vBbqBXCnsv8uU0UIfS1DBQ
**vDz46dvR6tEJoHtmYPcmBA**	Fondation Nicolas Hulot pour la Nature et l'Homme	https://www.youtube.com/channel/vDz46dvR6tEJoHtmYPcmBA
**vEp8zXBWqBgYnF4cK_ojlQ**	RFM France	https://www.youtube.com/channel/vEp8zXBWqBgYnF4cK_ojlQ
**vGQ0lxN0jn4Ia5l9FxnWUQ**	demaintv	https://www.youtube.com/channel/vGQ0lxN0jn4Ia5l9FxnWUQ
**vGiiF7ZvZAPPog9uwqlqtQ**	ThalassaOfficiel	https://www.youtube.com/channel/vGiiF7ZvZAPPog9uwqlqtQ
**vJ7MfsC_VxHQeYHAIo6Y6A**	Place Beauvau	https://www.youtube.com/channel/vJ7MfsC_VxHQeYHAIo6Y6A
**vMxyY86JXMMtzz0f-FAs5Q**	Moteur En Colère	https://www.youtube.com/channel/vMxyY86JXMMtzz0f-FAs5Q
**vRgiAmogg7a_BgQ_Ftm6fA**	Le Précepteur	https://www.youtube.com/channel/vRgiAmogg7a_BgQ_Ftm6fA
**vWV0HvOSGTchY-vym-tFaQ**	Philippe Bolo	https://www.youtube.com/channel/vWV0HvOSGTchY-vym-tFaQ
**vWagnsCJ9xY0EaP-kT7aUg**	DMZ TV	https://www.youtube.com/channel/vWagnsCJ9xY0EaP-kT7aUg
**vg4_wSz4Cmo4xRPXaKU47A**	C dans l'air	https://www.youtube.com/channel/vg4_wSz4Cmo4xRPXaKU47A
**vo4_caF8_r2olk-4BEvrKg**	Stupéfiant !	https://www.youtube.com/channel/vo4_caF8_r2olk-4BEvrKg
**voaoDVbNQtQNp9ZNUFNGLw**	Les Sentinelles du peuple	https://www.youtube.com/channel/voaoDVbNQtQNp9ZNUFNGLw
**w59BtkcwRwyveFgbF_UIRQ**	Adrien Morenas - Député de la 3ème circonscriptior	https://www.youtube.com/channel/w59BtkcwRwyveFgbF_UIRQ
**wDuF60hr22fza3T9Zkztxg**	Mayotte officiel	https://www.youtube.com/channel/wDuF60hr22fza3T9Zkztxg
**wI-JbGNsojunnHbFAc0M4Q**	ARTE	https://www.youtube.com/channel/wI-JbGNsojunnHbFAc0M4Q
**wLLr_Fo9dpdJ9UHq4A7PjA**	Révolution Permanente	https://www.youtube.com/channel/wLLr_Fo9dpdJ9UHq4A7PjA
**wLWjq0FcsNYNaWQI-WnIsw**	Culture Populaire	https://www.youtube.com/channel/wLWjq0FcsNYNaWQI-WnIsw
**wS6XOw-TiYb6eZsY9fgirA**	Agriculture innovante	https://www.youtube.com/channel/wS6XOw-TiYb6eZsY9fgirA
**wTvW0qRQXV-yvBghNVgqKg**	France 3 Nouvelle-Aquitaine	https://www.youtube.com/channel/wTvW0qRQXV-yvBghNVgqKg
**w_VR4FcJIotZlIdFwfkaxw**	Ça crève les yeux	https://www.youtube.com/channel/w_VR4FcJIotZlIdFwfkaxw
**wamokvn8ynE7Tp3oarOIFg**	Air Buzz	https://www.youtube.com/channel/wamokvn8ynE7Tp3oarOIFg
**we5Mx0spjObJrtUGRaCJWQ**	JEREMYSTERES	https://www.youtube.com/channel/we5Mx0spjObJrtUGRaCJWQ
**wi4cpbdkeuyYD8lDo3Z6qA**	Khaled Freak	https://www.youtube.com/channel/wi4cpbdkeuyYD8lDo3Z6qA
**wuSgFpWm1XHV8g7VSVHSwA**	Wheeler-Fisher	https://www.youtube.com/channel/wuSgFpWm1XHV8g7VSVHSwA
**x3LJBBmyKaUj3nDCY4KFRw**	Arti Bus	https://www.youtube.com/channel/x3LJBBmyKaUj3nDCY4KFRw
**x9ygIxPfYR05kY92KzvbvA**	Boulevard Voltaire	https://www.youtube.com/channel/x9ygIxPfYR05kY92KzvbvA
**xA8CClv0o7r-820LY7NOvA**	Gégé RVL777	https://www.youtube.com/channel/xA8CClv0o7r-820LY7NOvA
**xCAFgQPTZ-9Sj92-Q5Kmrg**	site de Jacques Chevalier	https://www.youtube.com/channel/xCAFgQPTZ-9Sj92-Q5Kmrg
**xE15Q9nQrELfOSgPzznyWw**	RP MEDIAS TV	https://www.youtube.com/channel/xE15Q9nQrELfOSgPzznyWw
**xEO_7FYNQG4_JqvGQIR_zQ**	Joachim Veliocas	https://www.youtube.com/channel/xEO_7FYNQG4_JqvGQIR_zQ
**xSTb76RvL18Se5PmglQA3g**	Sandrine Mörch	https://www.youtube.com/channel/xSTb76RvL18Se5PmglQA3g
**xUU_ol6UkF30-fPGb-taCw**	Alix a Toujours Raison VS Alixator Officiel	https://www.youtube.com/channel/xUU_ol6UkF30-fPGb-taCw
**x_92gSjC4jRvr2XVdBvWbQ**	Charlotte LECOCQ	https://www.youtube.com/channel/x_92gSjC4jRvr2XVdBvWbQ
**xc4L2LKEeViVyNA5taSBIA**	Canal Fi	https://www.youtube.com/channel/xc4L2LKEeViVyNA5taSBIA
**xi9qt3y4-L-DH253hH5j6Q**	Mad	https://www.youtube.com/channel/xi9qt3y4-L-DH253hH5j6Q
**xjWTS8fHnz10xO67jGryqw**	Ben on the road	https://www.youtube.com/channel/xjWTS8fHnz10xO67jGryqw
**xl36j3GZ56iHw47Nrctt2w**	Polynésie la 1ère	https://www.youtube.com/channel/xl36j3GZ56iHw47Nrctt2w
**xooxCiflBsJJ3PnGRg69jA**	Agriculture et Environnement	https://www.youtube.com/channel/xooxCiflBsJJ3PnGRg69jA
**xteEiPrio3px_iRV5VOnyw**	Institut Iliade	https://www.youtube.com/channel/xteEiPrio3px_iRV5VOnyw
**xuboKRCRvIBjeiwAEEe-Ow**	Caroline Janvier	https://www.youtube.com/channel/xuboKRCRvIBjeiwAEEe-Ow
**y02kaOc08UMChaU5U9qfGw**	Alexandra Louis	https://www.youtube.com/channel/y02kaOc08UMChaU5U9qfGw
**y6Iwt8U-1-y5_n9I0Sbe6g**	Armée de Terre	https://www.youtube.com/channel/y6Iwt8U-1-y5_n9I0Sbe6g
**y8Mth5GcW0oMwdc6We-Tlg**	Paris-Normandie	https://www.youtube.com/channel/y8Mth5GcW0oMwdc6We-Tlg
**y9rZHVP17n2Cxq3vGp1nKw**	Didier BAICHERE	https://www.youtube.com/channel/y9rZHVP17n2Cxq3vGp1nKw
**yAq3bU6g09JB0uaJkJyL8Q**	Xavier Roseren	https://www.youtube.com/channel/yAq3bU6g09JB0uaJkJyL8Q
**yIV8rkza5Uk_sJIhqilBvQ**	L'ÉQUIPE	https://www.youtube.com/channel/yIV8rkza5Uk_sJIhqilBvQ
**yJDHgrsUKuWLe05GvC2lng**	Stupid Economics	https://www.youtube.com/channel/yJDHgrsUKuWLe05GvC2lng
**yLTMnYGibxOe13Ph4Fcnbw**	Richard Lioger	https://www.youtube.com/channel/yLTMnYGibxOe13Ph4Fcnbw

**yN0QTIrTETHL1EgWvm1vJw**	Emmanuel et Sandrine	https://www.youtube.com/channel/yN0QTIrTETHL1EgWvm1vJw
**yNQkosbRl7ze1TFVUdZWLQ**	Le Parasite	https://www.youtube.com/channel/yNQkosbRl7ze1TFVUdZWLQ
**ySE3oPqVjBS5tiC8JYpZkg**	Jacky l'espoir	https://www.youtube.com/channel/ySE3oPqVjBS5tiC8JYpZkg
**yWLV25B3wAezyYMJOcTgoQ**	JAX OFFICIEL	https://www.youtube.com/channel/yWLV25B3wAezyYMJOcTgoQ
**yX-_BipdNpdFgeZEQBNstA**	Manon Aubry	https://www.youtube.com/channel/yX-_BipdNpdFgeZEQBNstA
**yZYClEbzAAx2IQuDIZSYWw**	CauseToujours	https://www.youtube.com/channel/yZYClEbzAAx2IQuDIZSYWw
**yamXi0qEQJghCjJbj__aWQ**	Chaîne officielle TVLibertés	https://www.youtube.com/channel/yamXi0qEQJghCjJbj__aWQ
**ycaROGreWZ5zjsn3s73pKQ**	QUINO-STUDIO TV	https://www.youtube.com/channel/ycaROGreWZ5zjsn3s73pKQ
**ynFUJ4zUVuh3GX7bABTjGQ**	Trouble Fait	https://www.youtube.com/channel/ynFUJ4zUVuh3GX7bABTjGQ
**yoauNI7m6L0ukiIf-2ermA**	Chasseur-de-Frissons	https://www.youtube.com/channel/yoauNI7m6L0ukiIf-2ermA
**yqiDJCxslvqiy4kw94BZfw**	Dérush	https://www.youtube.com/channel/yqiDJCxslvqiy4kw94BZfw
**z4z9r7Af8OqFu3cYfIR5hQ**	Kroc Blanc	https://www.youtube.com/channel/z4z9r7Af8OqFu3cYfIR5hQ
**zB4XvWgVlXFI4ljbQXzmwA**	Guillaume Deloison	https://www.youtube.com/channel/zB4XvWgVlXFI4ljbQXzmwA
**zBoVtUqSyAyr0kF5ABW7gQ**	RTL - On a tellement de choses à se dire	https://www.youtube.com/channel/zBoVtUqSyAyr0kF5ABW7gQ
**zKH70qfN_yuXq3s91fdwmg**	TV5MONDE Info	https://www.youtube.com/channel/zKH70qfN_yuXq3s91fdwmg
**zKNty_emTCKKeKkIkBfIVA**	SNCF	https://www.youtube.com/channel/zKNty_emTCKKeKkIkBfIVA
**zZiy3EANVAx7h2XYqXsVbw**	Collège de France	https://www.youtube.com/channel/zZiy3EANVAx7h2XYqXsVbw
**zbb9VE5NSBNXAPpou2kOVw**	CANAL+ Docs	https://www.youtube.com/channel/zbb9VE5NSBNXAPpou2kOVw
**zepcutq_PDtzpqJkfaueYw**	Jean-Paul Miniscloux	https://www.youtube.com/channel/zepcutq_PDtzpqJkfaueYw
**zjd9v6DMprAAvOBB4sxBPA**	LE GRAND JD	https://www.youtube.com/channel/zjd9v6DMprAAvOBB4sxBPA
**zkvdLzgFecbUBRdgPX3RLg**	Médias-Presse-Infos	https://www.youtube.com/channel/zkvdLzgFecbUBRdgPX3RLg
**zrjIzE1Hp3UkXAYEawK3JA**	Région Île-de-France	https://www.youtube.com/channel/zrjIzE1Hp3UkXAYEawK3JA
**zuTw_H26NjytroMxxeRc5Q**	Parti animaliste	https://www.youtube.com/channel/zuTw_H26NjytroMxxeRc5Q
**zwbuFRbTNv83QKTrgm1ndA**	L'histoire corrigée	https://www.youtube.com/channel/zwbuFRbTNv83QKTrgm1ndA
**zxHfZ_q7dV9PGQ9fh650nQ**	LA CAGOULE	https://www.youtube.com/channel/zxHfZ_q7dV9PGQ9fh650nQ
**zxgG5bX9Puo2aEJdozeg6A**	C l'hebdo	https://www.youtube.com/channel/zxgG5bX9Puo2aEJdozeg6A
